# The minimal important difference of patient-reported outcome measures related to female urinary incontinence: a systematic review

**DOI:** 10.1186/s12874-024-02188-4

**Published:** 2024-03-08

**Authors:** Jordana Barbosa-Silva, Letícia Bojikian Calixtre, Daniela Von Piekartz, Patricia Driusso, Susan Armijo-Olivo

**Affiliations:** 1 Women’s Health Research Laboratory (LAMU), Physical Therapy Department, Federal University of São Carlos, Rodovia Washington Luís, km 235, Monjolinho, São Carlos, SP 13565-905 Brazil; 2 Faculty of Business and Social Sciences, University of Applied Sciences – Hochschule Osnabrück, Osnabrück, Germany; 3 Universidade de Pernambuco, Campus-Petrolina, Brazil; 4 Faculty of Rehabilitation Medicine/Faculty of Medicine and Dentistry, University of Alberta, Edmonton, Canada

**Keywords:** Clinical significance, Minimal clinically important difference, Minimal important difference, Patient-reported outcomes (PROMs), Urinary incontinence, Women’s health

## Abstract

**Background:**

The minimal important difference is a valuable metric in ascertaining the clinical relevance of a treatment, offering valuable guidance in patient management. There is a lack of available evidence concerning this metric in the context of outcomes related to female urinary incontinence, which might negatively impact clinical decision-making.

**Objectives:**

To summarize the minimal important difference of patient-reported outcome measures associated with urinary incontinence, calculated according to both distribution- and anchor-based methods.

**Methods:**

This is a systematic review conducted according to the PRISMA guidelines. The search strategy including the main terms for urinary incontinence and minimal important difference were used in five different databases (Medline, Embase, CINAHL, Web of Science, and Scopus) in 09 June 2021 and were updated in January 09, 2024 with no limits for date, language or publication status. Studies that provided minimal important difference (distribution- or anchor-based methods) for patient-reported outcome measures related to female urinary incontinence outcomes were included. The study selection and data extraction were performed independently by two different researchers. Only studies that reported the minimal important difference according to anchor-based methods were assessed by credibility and certainty of the evidence. When possible, absolute minimal important differences were calculated for each study separately according to the mean change of the group of participants that slightly improved.

**Results:**

Twelve studies were included. Thirteen questionnaires with their respective minimal important differences reported according to distribution (effect size, standard error of measurement, standardized response mean) and anchor-based methods were found. Most of the measures for anchor methods did not consider the smallest difference identified by the participants to calculate the minimal important difference. All reports related to anchor-based methods presented low credibility and very low certainty of the evidence. We pooled 20 different estimates of minimal important differences using data from primary studies, considering different anchors and questionnaires.

**Conclusions:**

There is a high variability around the minimal important difference related to patient-reported outcome measures for urinary incontinence outcomes according to the method of analysis, questionnaires, and anchors used, however, the credibility and certainty of the evidence to support these is still limited.

**Supplementary Information:**

The online version contains supplementary material available at 10.1186/s12874-024-02188-4.

## Introduction

The International Continence Society defines urinary incontinence as any loss of urine [1]. Stress urinary incontinence has been defined as urine loss associated with coughing, sneezing, exertion, or physical exertion; while urgent urinary incontinence is defined as loss of urine associated with urinary urgency (a sudden and strong urge to urinate) and mixed urinary incontinence combines both stress and urge incontinence, concomitantly [1].

According to the World Health Organization, urinary incontinence affects more than 200 million people worldwide [2, 3] being more prevalent in women [4]. One in four women will be incontinent at some point in life [4, 5]. The high prevalence of urinary incontinence concerns government institutions, as the costs related to urinary incontinence care are high, varying from around 117 million and $66 billion (2007 US dollars) per year in the United Kingdom [6] and the United States of America [7], respectively. The consequences of urinary incontinence are associated with impairment of social, psychological, financial, and sexual aspects of a woman’s life. This in turn can be related to reduced quality of life [8], self-esteem, and social isolation [9]. Moreover, urinary incontinence is a predictor of mortality, especially among the elderly [10].

Patient-reported outcome measures and voiding diaries are used to measure the quality of life of patients with urinary incontinence, as well as to quantify urinary loss. In both clinical practice and research, patient-reported outcome measures are useful for reporting the effects of interventions since they take into consideration the patients’ perspective regarding the changes observed after the treatment. However, the interpretation of scientific research results in general looks mainly at the interpretation of statistical analyses, that is, whether the result of any intervention may or may not be considered statistically significant [11]. The sole interpretation of the “p” values is insufficient to demonstrate the impact of the intervention on the health care of individuals [12, 13], as sometimes the research findings may be statistically significant but cannot be considered clinically relevant, as the patient did not have a clinically significant improvement [14].

The analysis of clinical significance has increasingly been used in health research, enabling it to attest to whether the result from a treatment is perceived as beneficial by the patient or any stakeholder’s perspective [15]. One of the methods used to help with the interpretation of the clinical relevance of research results is the use of the minimal important difference of clinical outcome measures. The minimal important difference has been defined as “the smallest difference in score in the domain of interest that patients perceive as important, either beneficial or harmful, and which would lead the clinician to consider a change in the patient’s management’’ [16].

There are two different methods to determine the minimal important difference: [17] (1) Distribution methods use statistical calculations based on the distribution of outcomes scores to determine how the scores differ among patients [18]. Although these methods are easily applied, they do not evaluate the clinical relevance of the intervention according to the patient's perception [16]. (2) Anchor-based methods take into consideration patients’ perceptions by using interpretive and self-reported tools such as the global rating of change scale [19–22] for assessing change in the outcome, which represents a meaningful degree of change [23]. In this case, the patient has the autonomy to add a numerical value to the status of the main complaint, considering their perception. Psychosocial factors, for example, could potentially influence the patient's global status, which may interfere with the variable of interest [16].

Previous systematic reviews have assessed the minimal important difference for outcomes related to the musculoskeletal [24–26] and oncological [27] areas but none of them have focused on evaluating minimal important difference for outcomes related to urinary incontinence, which has a negative impact on this research field, as it impairs the estimation of sample sizes and the interpretation of the results of clinical trials. This lack in the literature may directly affect the over- or underestimation of the clinical significance of studies that have already been published or will be in the future. In addition, the lack of clear guidance on how to interpret the clinical relevance of results from urinary incontinence outcomes does not contribute to evidence-based practice [28]. Synthesizing the evidence about the clinical relevance of instruments related to urinary incontinence may benefit clinicians and researchers, [29] improving decision-making, by informing the minimal important difference of specific instruments, which may be listed in clinical and scientific practice [30].

Therefore, the aims of the present systematic review were: I) to identify and synthesize all distribution-based and anchor-based methods to estimate minimal important difference for outcome measures related to urinary incontinence; II) to summarize minimal important difference estimates related to the most commonly used outcome measures related to urinary incontinence; III) to determine the credibility of minimal important difference reported in each study.

## Methods

This is a systematic review conducted according to the PRISMA [31] and COnsensus-based Standards for the selection of health Measurement INstruments [32] guidelines and registered in PROSPERO (protocol CRD42022299686).

### Eligibility criteria, information sources, search strategy

The inclusion and exclusion criteria were based and adapted according to the PICOs and COSMIN frameworks, as described below:

*Population*: Women older than 18 years old, with stress, urge and/or mixed urinary incontinence according to International Continence Society definitions(1); with diagnostic of urinary incontinence according to the results of a subjective or objective assessment. Studies were excluded if the aim was to analyze urinary symptoms of children or men; if they included only continent women and/or if authors analyzed only other pelvic floor dysfunctions (i.e., fecal and/or anal incontinence, pelvic organ prolapse, sexual dysfunctions).

*Intervention/Instruments of interest (construct targeted)*: Studies were included if they assessed any outcome measure related to urinary incontinence, such as quality of life and/or amount of leakage. We also looked for outcomes that assessed pelvic floor muscles function evaluated through by questionnaires or physical tests that include vaginal palpation, dynamometry, vaginal cones, manometry, electromyography, imaging exams, urodynamic and/or urine stream interruption test [33]. However, no studies were found during screening.

*Comparison:* Not applicable.

*Outcomes:* Studies that reported minimal important differences that could be derived from distribution- or anchor-based methods as described in a previous study [17] were included. A detailed description of the methods available to determine minimal important difference in clinical research are presented in Appendix 1.

*Study design:* Any study generating minimal important differences for urinary incontinence outcomes (randomized control trials and controlled trials, secondary analysis of clinical trials, cohort studies, cross-sectional studies, reliability, responsiveness, and validity studies) were included. The following types of studies were excluded: case reports, reviews, systematic reviews, meta-analyses, commentaries, letters to the editor, conference papers, books chapter, protocol registration, abstracts without full text, and experimental studies. Reviews were carefully looked for relevant references.

Searches were performed in June 09 2021 and updated in January 09 2024, including the main terms for urinary incontinence and minimal important difference. In addition, a search filter focusing on clinical significance keywords obtained from previous publications was used [34] (details available in Appendix 2). Five databases were consulted: Medline (Ovid MEDLINE(R) ALL), Embase (Ovid interface), CINAHL PLUS with Full text (EBSSCOhost interface), Web of Science (Indexes=SCI-EXPANDED, SSCI, A&HCI, ESCI) and Scopus. No limits were applied for the date, language, or publication status. A manual search was performed to look for relevant references. Included studies were tracked with the web of Sciences database.

### Study selection

Results from searchers were compiled into ENDNOTE software and imported to Covidence (www.covidence.org), which was used during the screening process. Two independent researchers evaluated the studies' eligibility according to the inclusion and exclusion criteria in two sequential evaluation phases: (I) analysis of titles and abstracts; and (II) analysis of full texts. In case of disagreement, a consensus meeting was performed. In any case of continuous discrepancy, a third evaluator makes the final decision. The PRISMA flowchart [35] was provided with the results of the selection process.

### Data extraction

An Excel form was developed for data extraction. Pilot testing and regular revision through discussions were taken to standardize the data extraction form and process. One researcher conducted the data extraction and organized the data on the Excel form and a second researcher reviewed the extracted data for accuracy and completeness. Disagreements were solved in consensus meetings.

Data extracted was based on characteristics that include, but were not limited to: 1) article information (first author, year of publication, language, funding, country, aims, study design, and setting); 2) population information (age, diagnosis, tool for the diagnosis and other conditions or characteristics); 3) outcome measurements (minimal important difference determination (e.g. analytical approach, sample size, duration of follow-up when applicable); minimal important difference estimation methods (distribution- and/or anchor-based; the specific anchor applied during data collection, minimal important difference values); constructs evaluated (e.g. quality of life evaluated according to patient-reported outcome measures, pelvic floor function, urinary loss); tool description (categorical, ordinal, or numerical data); type of outcome (patient-reported outcome measures or physical test)); 4) summary of results (minimal important difference estimation, correlations between the outcome and anchor, precision of the minimal important difference (e.g. 95% confidence interval/ minimal important difference *100), time between baseline and follow-up, directions of both anchor and patient-reported outcome measures (e.g., if the increase of scores of both instruments reflect an improvement, worsened, or if the scores from both instruments have opposite meaning), correlations of the patient-reported outcome measures and the transition item during baseline and follow-up). In case of missing quantitative data, the authors of the primary studies were contacted in order to get unreported data. When the authors did not answer our request, data were extracted from the graphs available in the studies.

### Credibility of minimal important difference estimates

Two independent researchers conducted the credibility assessment of the minimal important difference in each included study that used anchor-based methods. As far as the authors' knowledge, there is no specific tool to assess the credibility of minimal important differences reported according to distribution-based methods. The credibility was evaluated separately for each minimal important difference by two assessors and the final assessment was determined after a consensus meeting between the two reviewers. The instrument developed by Devji et al. [34] for this specific purpose was used under license authorization from McMaster University, as it is the only published tool created for evaluating the credibility of the minimal important difference generated by anchor-based methods. It is composed of 1) a core criterion with five items related to anchor-based methods, and 2) four items related to the transition rating anchors. The first item has a dichotomic yes/no response option, however, the other items from the instrument are composed by a five-point scale with the following response options: definitely yes, to a great extent, not so much, definitely no, or impossible to tell.

There is no specific guidance on how to summarize different domains of this tool as a final assessment of the credibility of the minimal important difference. Therefore, the final assessment for each minimal important difference was defined according to previous decision rules prepared by the team, to create three different categories of credibility: these were based on similar decision rules used when implementing the Cochrane risk of bias (RoB2) tool for randomized controlled trials. Three different categories were created to determine the final assessment of minimal important difference credibility as follows:

1. Low credibility: when most part or one of the items was scored with a negative answer (i.e., not so much or definitely no);
2. Some concerns: when no negative answers were assessed, and the rest of the questions were assessed as “impossible to tell”;
3. High credibility: when all the questions were assessed with a positive answer (i.e., to a great extent or definitely yes).

### Data synthesis

The findings of this review were described in a narrative (descriptive) synthesis, organized in evidence tables that compiled study details, results, and data analysis. Data synthesis was performed according to the patient-reported outcome measures reported by the authors and the method of calculation for providing the minimal important difference. Minimal important difference provided by distribution-based methods were analyzed separately according to the type of calculation (i.e., effect size, standardized response mean, standard error of measurement, standard deviation) and time range of re-evaluation (e.g., 6 weeks, 12 weeks, 12 months). minimal important difference provided by anchor-based methods were performed following guidance from a previous systematic review about minimal important difference [26]. The absolute minimal important difference (mean difference associated with minimum improvement) was calculated for each study separately by checking the original papers and by extracting the mean change of the group of participants that reported a slight improvement, according to the anchor applied during data collection.

After data synthesis, we planned to plot all minimal important difference estimates based on anchor methods together by triangulation, in order to define a single value for each instrument included in the present review, considering that we would find evidence from multiple studies. However, the primary studies presented a high heterogeneity considering patient-reported outcome measures, anchors, and population characteristics, which violated the recommendations to perform the triangulation [36]. Also, a meta-analysis was not possible to conduct because of insufficient data.

### Quality of evidence

The Grading of Recommendations Assessment, Development, and Evaluation (GRADE) [37] approach was applied in order to assess the overall certainty of the evidence and to grade the strength of recommendations from minimal important differences reported according to anchor-based methods. This assessment was based on the credibility of the minimal important difference (that was analog to the risk bias of studies), inconsistency, indirectness, imprecision, and publication bias. We reported GRADE following previous recommendations on how to rate the certainly of evidence in the absence of pooled results and meta-analysis [38].

The level of evidence was downgraded for inconsistency and/or indirectness in cases where: minimal important differences from patient-reported outcome measures were reported by a single study; different anchors were applied in order to calculate the minimal important difference, studies included different population diagnoses or time-points when the minimal important differences were calculated; studies used different levels of improvement to determine the minimal important difference (minimal, moderate, or strong) when conducting their analysis. The imprecision was downgraded when the total sample size population was less than 300 participants.

The final rating of the studies was classified as high, moderate, low, or very low certainty of evidence [37].

## Results

### Study selection

**Fig. 1:**
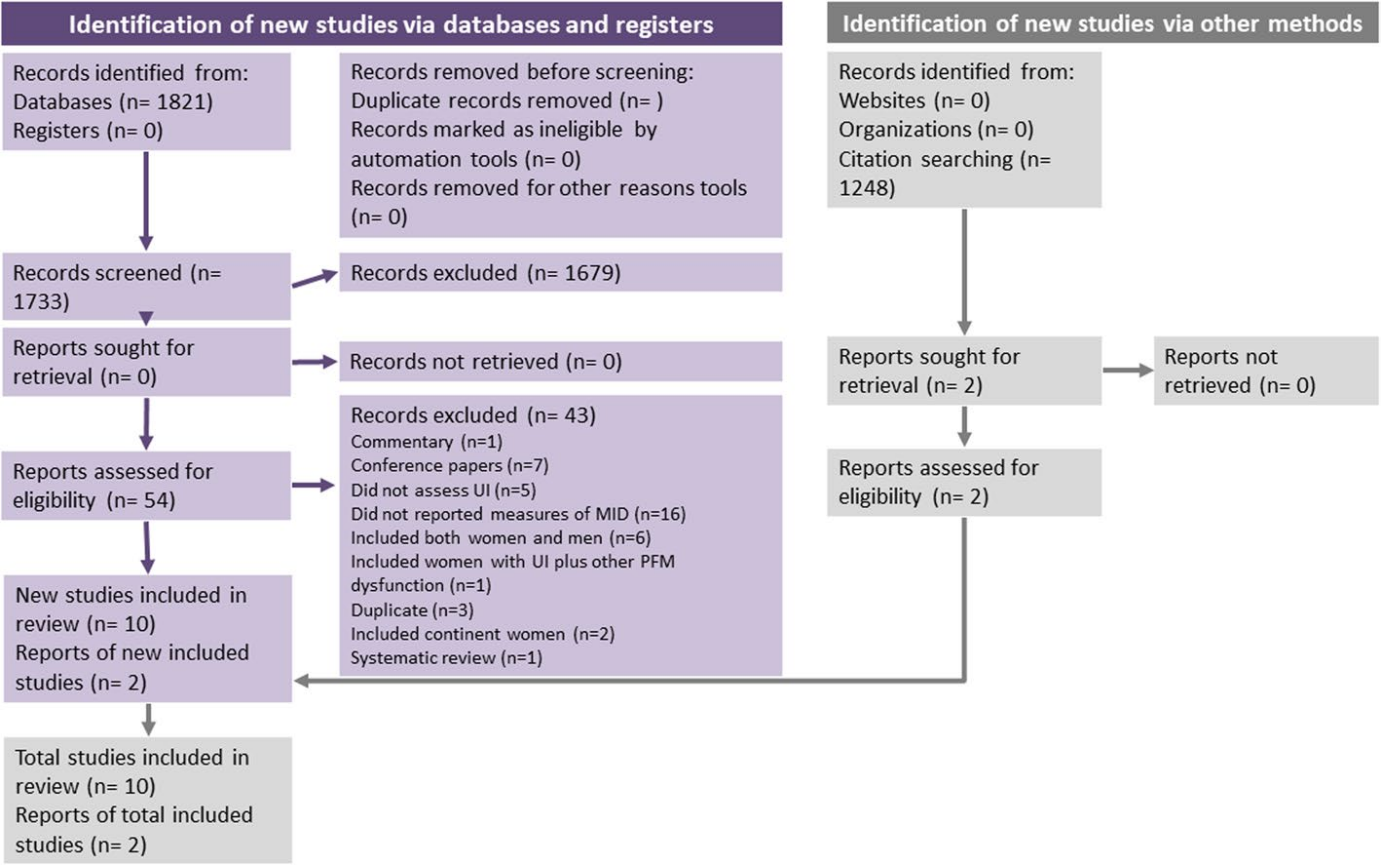
PRISMA flowchart

A total of 1,662 papers were found through the database search, 719 references were duplicated, so the final number of studies included in the data screening was 943. According to the screening of titles and abstracts, 54 potential studies were selected for full-text review and 10 studies met the inclusion criteria [39–48]. Reasons for exclusion are available in the PRISMA flowchart (Fig. 1) and details of exclusions are provided in Appendix 3. After the manual search, two additional studies were included [49, 50]. Therefore, 12 studies were analyzed.

### Characteristics of included studies

The general information of the 12 studies included in the study is described in Table 1. Most of the studies were conducted in the United States of America [39–42, 44, 46], and published after 2010 [42–50], and minimal important differences were derived mainly from data of randomized controlled trials [39–42, 46, 48, 50], related to non-surgical [39–42, 45, 48, 50] and surgical [43, 44, 46, 47, 49, 50] interventions. One study conducted as a secondary analysis from two different trials assessed the surgical and conservative effectiveness of UI interventions [50]. Nine studies included participants with stress urinary incontinence [40, 41, 43–46, 48–50], one study included participants with urgency stress urinary incontinence [42] and three included women with mixed stress urinary incontinence [39, 47, 50]. The diagnosis of the participants’ symptoms was assessed by subjective (i.e., self-reported, validated questionnaires, health professionals interviews) and objective tools and tests, specially by urodynamics. Eight studies reported minimal important differences according to distribution-based methods [41–44, 46–49], while 10 studies reported minimal important difference according to anchor-based methods [39–43, 45, 46, 48–50].

**Table 1:** General information of included studies (n=12)

Studies characteristics	n (%)		
Country		**Study setting**	
United States of America	6 (50.0)	Clinic	2 (16.6)
China	1 (8.3)	Hospital	2 (16.6)
Portugal	1 (8.3)	Multicenter	7 (58.3)
Malysia	1 (8.3)	Online survey with online intervention	1 (8.3)
Sweden	1 (8.3)		
Germany	1 (8.3)		
United Kingdom	1 (8.3)		
Language		**Published date**	
English	12 (100)	Before 2000	1 (8.3)
		Between 2000 and 2010	2 (16.6)
		After 2010	9 (75)
Study Design		**Type of intervention**	
Randomized controlled trial	8 (66.6)	Non-surgical	6 (50.0)
Clinical trial	1 (8.3)	Surgical	5 (41.6)
Longitudinal	1 (8.3)	Surgical and non-surgical interventions	1 (8.3)
Cross-sectional	2 (16.6)		
Diagnosis tool		**Condition**	
Interview with a urotherapist	2 (16.6)	Stress urinary incontinence	10 (83.3)
Self-reported	3 (25)	Urgency urinary incontinence	1 (8.3)
Validated questionnaire	3 (25)	Mixed urinary incontinence	3 (25)
Voiding diary	2 (16.6)		
Cough test	4 (33.3)		
Pad-test	1 (8.3)		
Urodynamics	4 (33.3)	**Methods to report minimal important difference**	
Algorithm	1 (8.3)	Distribution-based methods	8 (66.6)
Uroflowmetry	1 (8.3)	Anchor-based methods	10 (83.3)
Cystometry	1 (8.3)		
Not reported	3 (25)		
Patient Report Outcomes (PROMs)	n (%)	**Anchors**	n (%)
Australian Pelvic Floor Questionnaire	1 (8.3)	Global Perception of Improvement (GPI)	1 (8.3)
Incontinence Impact Questionnaire (IIQ)	1 (8.3)	Incontinence Impact Questionnaire (IIQ)	1 (8.3)
Incontinence Quality of Life (I-QOL)	2 (16.6)	Incontinence Severity Index	1 (8.3)
International Consultation on Incontinence Questionnaire - Short Form (ICIQ-SF)	3 (25)	Pad test	2 (16.6)
ICIQ-Lower Urinary Tract Symptoms Quality of Life (ICIQ-LUTSqol)	1 (8.3)	Patient Global Impression of Improvement questionnaire	8 (66.6)
Kings Health Questionnaire (KHQ)	1 (8.3)	Patient Satisfaction Questionnaire (PSQ)	1 (8.3)
Michigan Incontinence Symptom Index (M-ISI)	1 (8.3)	Self-reported about the satisfaction with the treatment	3 (25)
Overactive Bladder Questionnaire (OAB-q)	1 (8.3)	Voiding diary	5 (41.6)
Urinary Impact Questionnaire (UIQ)	3 (25)	Urogenital Distress Inventory (UDI)	1 (8.3)
Urogenital Distress Inventory (UDI)	3 (25)	10-cm Visual Analogue Scale indicating the severity of symptoms	1 (8.3)
Urogenital Distress Inventory (UDI-irritative symptoms)	1 (8.3)		
Urogenital Distress Inventory (UDI-stress)	1 (8.3)		
International Consultation on Incontinence Questionnaire – Female Lower Urinary Tract Symptoms (ICIQ-FLUTS)	1 (8.3)		

### Analysis of credibility

Ten studies [39–43, 45, 46, 48–50] determined minimal important differences of several patient-reported outcome measures using anchor-based methods and provided 78 different minimal important differences. Therefore, we performed one evaluation for each minimal important difference separately, resulting in 78 credibility assessments. All reports related to minimal important differences according to anchor-based methods presented low credibility. More details about the scores of the credibility tool are reported in Appendix 4.

In most cases (n=78), the studies met the first criterion of the tool, that assesses if participants responded to the patient-reported outcome measures and the anchor directly. Moreover, anchors used during data collection were considered understandable (second criteria) in 75 cases.

In 24 derived minimal important difference calculations, the correlation between the patient-reported outcome measures and the anchor was not reported (third criteria), although most authors mentioned a general correlation of ≥0.3 between the instruments (n=52). Similarly, most authors failed to meet the fourth criteria of the tool that measured the precision estimate of the minimal important difference (n=61; 78.2%). In 42 cases, the criterion applied by the anchor did not reflect a small but important difference between the health status of the patients, which contradicts the definition of the minimal important difference.

For 63 minimal important difference estimates, the range of time between the first and the second assessments was considered long (more than two or three months); which is the sixth criteria. This can likely be linked to recall bias (i.e., biased perception of the actual health(34)) and difficulty in assessing the previous health status [34]. The correlation between the transition score and the prescore and postscore on the target instrument (seventh and eighth criteria) was reported only in few estimates in three different studies [42, 43, 46].

The risk of bias graph and the summary results are presented in Appendix 5 and 6, respectively.

### Synthesis of results

All minimal important difference estimates were provided for 13 different patient-reported outcome measures. Although we targeted several types of outcomes in this review, no study reported minimal important difference estimates for physical assessment of pelvic floor muscles’ function, for example. Some authors also provided the minimally important difference for subscales of patient-reported outcome measures. This was the case for the Incontinence Quality of Life (I-QOL): Avoidance and Limiting Behavior, Psychosocial Impacts and Social Embarrassment domains [40]; Pelvic Floor Impact Questionnaire (PFIQ) – UIQ subscale; Pelvic Floor Distress Inventory (PFDI) – general score for UDI [43], and stress and irritative subscales [41]; Overactive Bladder Questionnaire (OAB-q) – Symptom Severity subscore [42]; the Australian Pelvic Floor Questionnaire – Bladder and global score [49]; and the International Consultation on Incontinence Questionnaire – Female Lower Urinary Tract Symptoms (ICIQ-FLUTS) – incontinence domain [50].

Ten different subjective and objective anchors were found among the studies. The Patient Global Impression of Improvement also known as the Global Rating Scale was the most used, followed by the voiding diary, satisfaction with the treatment, and the pad test.

Table 2 describes the main details regarding the population, the patient-reported outcome measures, anchors, data analysis, and conclusions reported by the included studies. Although one study reported minimal important differences according to anchor methods for the Michigan Incontinence Symptom Index (M-ISI) [44], results were not considered in the present review because the statistical method applied by the authors was not clear in the manuscript, and the authors did not respond our e-mail. Appendix 7 provides details about the methods and concepts used to provide minimal important differences using anchor-based methods. Appendix 8 presents a matrix table with a compilation of the minimal important differences extracted from the primary studies according to the distribution and anchor-based methods.

**Table 2:** Characteristics of primary studies included in this systematic review

Author	Objective	Sample size	Diagnosis Treatment	PROM	Distribution analysis	Anchor based-analysis	Anchor	Nº of MIDs	Conclusions
Patrick et al., 1999 [39]	To report the further development of the I-QOL, including its measurement model, responsiveness, and effect size	Start: 288 End: The calculations for each anchor had a different sample size: **Pad test:** 270 **Voiding diary**: 269 **PGI-I**: 115	SUI, MUI Conservative: pharmacological	I-QOL (0-100 points; higher score means a worse condition)	NA	Responsiveness statistic (mean change divided by the stadard deviation of the baseline)	PGI-I, Pad test, Voiding diary	9	Results varied from 0.4.-0.8 and a varying from 2-13% was detected by the PROM, which supports the ability of the PROM to detect change.
Yalcin et al., 2005 [40]	To determine MID for the total and subscale I-QOL scores during within- and between-treatment for women with SUI	Start: Total=1133 Placebo=45 Treatment=708 End: Not reported	SUI Conservative treatment: pharmacological and placebo	I-QOL (0-100 points; higher score means a worse condition)	NA	Mean change	PGI-I	8	The clinical important score por I-QOL for reasons of between-treatment is 2.5 points, while the within-treatment score is 6.3
Barber et al., 2009 [41]	Estimate MID for the UDI, UDI-stress (subscale of the PFDI), and UIQ (subscale of the PFIQ)	Start: 445 End: 333	SUI Conservative treatment: pessary, behavioral therapy	UDI (0-300 points; higher score means a worse condition) UDI-stress, (0-100 points; higher score means a worse condition) UIQ (0-100 points; higher score means a worse condition)	Effect size, Standard error of measurement	Mean change	PGI-I, Incontinence Severity Index, Voiding diary	21	MIDs were provided for UDI, UDI-stress subscale and UIQ. Findings that meet or exceed these thresholds should be considered clinically important
Dyer et al., 2011 [42]	To estimate the MID for the UDI, IIQ, OAB-q and/or their selected subscales in patients with UUI and to determine whether the MID changes over time	Start: 307 End: 10 weeks = 272 8 months = 241	UUI Conservative: pharmacological, behavioral therapy	IIQ (0-400 points, higher score means a worse condition) OAB-q (0-100 points; higher score means a worse condition) UDI (0-300 points; higher score means a worse condition)	Effect size, Standard error of measurement	Difference in the mean score between people that were “better” versus “about the same”	PSQ, GPI, Voiding diary	38	MIDs were provided for women who underwent treatment for UUI in UDI, UDI irritative and OAB-q (quality of life and symptom severity subscores)
Chan et al., 2013 [43]	To evaluate the responsiveness of the Chinese PFDI and PFIQ in women with POP and/or urodynamic SUI who were undergoing treatment by surgery	Start: Continence surgery alone: 28 End: Not reported	SUI Non-conservative: surgery	UDI (subscale of PFDI) (0-300 points; higher score means a worse condition) UIQ (subscale of PFIQ) (0-100 points; higher score means a worse condition)	Effect size, Standardized response mean	Mean change	Satisfaction with the treatment received, 10-cm VAS score indicating the severy of symptoms	28	The Chinese version of the PFDI and PFIQ are responsive to change in incontinent women treated by surgical procedures
Suskind et al., 2014 [44]	To develop a clinically relevant, easy to use, and validated instrument for assessing severity and bother related to urinary incontinence	Start: 447 End: 447	SUI Non-conservative: surgery	M-ISI (1-12 points: higher score means a worse condition)	Standard deviation	NA	NA	8	M-ISI is a parsimonious measure that has established reliability and validity on several levels and complements current clinical evaluative methods for patients with urinary incontinence
Nystrom et al., 2015 [45]	To calculate the MID for ICIQ-UI SF and ICIQ-LUTSqol in women treated with PFMT for SUI	Start: 218 End: 214	SUI Conservative: Internet-based treatment programme (information about SUI and associated lifestyle factors, PFMT, training reports)	ICIQ-SF (0-21 points; higher score means a worse condition) ICIQ-LUTSqol (19-76 points; higher score means a worse condition)	NA	Mean change	PGI-I	4	This study suggested that the reductions of 2.5 and 3.7, should be considered clinically relevant for ICIQ-UISF and ICIQ-LUTSqol, respectively, in women with SUI treated with PFMT by internet or postal treatment
Sirls et al., 2015 [46]	To determine the MID of the ICIQ-UI SF	Start: 597 End: 12 months: 501 24 months: 447	SUI Non-conservative: surgery	ICIQ-SF (0-21 points; higher score means a worse condition)	Effect size	Mean change	PGI-I, UDI, IIQ, Voiding diary, Satisfaction with surgical results	14	The recommended MIDs for the ICIQ-UI SF in a population of women with stress predominant UI are -5 for assessment at 12 months and -4 for assessment at 24 months
Luz et al., 2017 [47]	To calculate KHQ scores for subjective cure and improvement rates	Start: 204 End: 6 months: objective=199; KHQ=190 12 months: objective=185, KHQ=177 24 months: objective=171; KHQ=169	SUI, MUI Non-conservative: surgery	KHQ (0-100 points; higher score means a worse condition)	Effect size	NA	NA	1	This study determined the clinically relevant threshould scores to define subjective outcomes after surgery for urinary symptoms
Baessler et al., 2019 [49]	To establish the MID of the Australian Pelvic Floor Questionnaire in women undergoing surgery for SUI or POP using anchor-based methods	Start: 80 End: 80	SUI Non-conservative: surgery	Australian Pelvic Floor Questionnaire (0-40 points; higher score means a worse condition)	Effect size, Standardized response mean	ROC curve	PGI-I	15	Changes of approximately 1 in the Australian Pelvic Floor Questionnaire can be considered a clinically important difference
Lim et al., 2019 [48]	To estimate the MIDs of ICIQ-UI SF and the ICIQ-LUTSqol using anchor and distribution-based methods for women with SUI undergoing nonsurgical treatment	Start: 120 End: 106	SUI Conservative: magnetic stimulation	ICIQ-SF (0-21 points; higher score means a worse condition) ICIQ-LUTSqol (19-76 points; higher score means a worse condition)	Effect size	Mean difference	PGI-I, Satisfaction with the treatment, 1-h pad test, Voiding diary (3-day bladder diary)	10	Reductions in 4 and 6 points at 12-month follow-up in ICIQ-UI SF and ICIQ-LUTSqol are perceived as clinically meaningful in women undergoing nonsurgical treatment for SUI
Nipa et al., 2023 [50]	To establish, for the first time, the clinically important differences for the ICIQ-UI-SF and ICIQ-FLUTS questionnaires following surgical and conservative treatments for stress-predominant urinary incontinence in women.	Start: 1200 End: 912	SUI, MUI Non-conservative: surgery Conservative: PFMT versus PFMT plus biofeedback	ICIQ-SF (0-21 points; higher score means a worse condition) ICIQ-FLUTS (0-20 points: higher score means a worse condition)	NA	Linear combination of the interaction and the variable indicating a change on the anchor variable	PGI-I	13	

*GPI* Global Perception of Improvement, *ICIQ-FLUTS* International Consultation on Incontinence Questionnaire – Female Lower Urinary Tract Symptoms, *ICIQ-SF* International Consultation on Incontinence Questionnaire – Short Form, *ICIQ-LUTSqol* ICIQ-Lower Urinary Tract Symptoms Quality of Life, *IIQ* Incontinence Impact Questionnaire, I-QOL: Incontinence Quality of Life, *KHQ* King’s Health Questionnaire, *MID* Minimal important difference, *M-ISI* Michigan Incontinence Symptom Index, *MUI* Mixed urinary incontinence n: sample size, *nº* Number, *OAB-q* Overactive Bladder Questionnaire, *PFMT* Pelvic floor muscles training, *PGI-I* Patient Global Impression of Improvement, *PSQ* Patient Satisfaction Questionnaire, *POP* Prolapse organ pelvic, *PROM* Patient-reported outcome measure, *ROC* Receiver operating characteristic, *SUI* Stress urinary incontinence, *UDI* Urogenital Distress Inventory, *UDI-stress* Urogenital Distress Inventory, stress symptoms subscale, *UIQ* Urinary Impact Questionnaire, *UUI* Urgency urinary incontinence, *VAS* Visual analogue scale

Tables 3 and 4 provide the qualitative data extracted from the studies that reported minimal important differences according to distribution- and anchor-based methods, respectively. Minimal important difference estimates for distribution-based methods represent the “points” for each patient-reported outcome measure. Three main distribution-based analyses were used by the included studies: effect size, standardized response mean, and standard error of measurement. For minimal important difference reported according to anchor method, it was reported by different estimates, including the mean, standard deviation, and absolute value, followed by the 95% confidence intervals and minimum-maximum values for the specific patient-reported outcome measures. Time points (follow-up) were different between studies (6, 10, 12, 14 weeks; and 4, 8, 12 and 12 months). In addition, there was a lack of clarity regarding the time point in four primary studies [42, 44, 46, 47]. Table 4 also shows the level of improvement considered by the authors when calculating the minimally important differences by anchor-based methods according to different symbols. Although different patient-reported outcome measures and anchors were applied, most of the studies did not consider the smallest difference identified by the participants to calculate the minimal important difference. The most used level to generate the minimal important difference was moderate to strong improvement.

**Table 3:** Quantitative results from the studies included in the present systematic review, according to distribution-based methods.

Analysis	PROM	Follow-up	Total score or domains	Power	MID^b^
Effect size^a^	Australian Pelvic Floor Questionnaire	6 weeks [49]	Bladder score	Authors did not specify if the effect size was small, medium or high	1.5
			Global score		1.2
	Incontinence Impact Questionnaire (IIQ)	Unclear [42]	Total score	0.2	-19.9
				0.5	-49.7
	International Consultation on Incontinence Questionnaire - Short Form (ICIQ-SF)	Unclear [46]	Total score	0.2	-0.82
				0.5	-2.05
		52 weeks (12 months) [48]	Total score	0.5	1.7
	International Consultation on Incontinence Questionnaire -Lower Urinary Tract Symptoms Quality of Life (ICIQ-LUTSqol)	52 weeks (12 months) [48]	Total score	0.5	5.2
	King’s Health Questionnaire (KHQ)	Unclear [47]	Total score	0.8	10
	Michigan Incontinence Symptom Index (M-ISI)	Unclear [44]	Total score	0.2	4.53
			Subscore: SUI		1.79
			Subscore: UUI		2.04
			Subscore: Pad use		1.19
			Total score	0.5	3.02
			Subscore: SUI		1.19
			Subscore: UUI		1.36
			Subscore: Pad use		0.79
	Urogenital Distress Inventory (UDI)	12 weeks [41]	Total score	0.2	−8.1 (−8.8, −7.5)^c^
		12 weeks [43]			-6
		Unclear [42]			-9.9
	Urogenital Distress Inventory (UDI)	12 weeks [41]	Total score	0.5	−20.5 (−18.8, 21.9)^c^
		12 weeks [43]			-16
		Unclear [42]			-24.8
	UDI-Subscale	Unclear [42]	Irritative subscale	0.2	-4.4
				0.5	-10.9
	UDI-Subscale	12 weeks [41]	Stress subscale	0.2	−3.9 (−4.2, −3.6)^c^
				0.5	−9.8 (−9.1, −10.6)^c^
	Urinary Impact Questionnaire (UIQ)	12 weeks [41]	Total score	0.2	−11.5 (−12.4, 10.7)^c^
				0.5	28.7 (−26.7, 31.1)^c^
		12 weeks [43]		0.2	-17
				0.5	-42
	Overactive Bladder Questionnaire (OAB-q)	Unclear [42]	Total score	0.2	4.8
				0.5	12.1
	OAB-q: subscale	Unclear [42]	Symptom severity	0.2	-4.2
				0.5	-10.4
Standardized response mean	Australian Pelvic Floor Questionnaire	6 weeks [49]	Bladder score	NA	1.4
			Global score		1.3
Standard error of measurement	Incontinence Impact Questionnaire (IIQ)	Unclear [42]	Total score	NA	-18.2
	Urogenital Distress Inventory (UDI)	12 weeks [41]	Total score	NA	−15.3 (−14.2, 16.4)^c^
		12 weeks [43]			-11
		Unclear [42]			-22.1
	UDI-Subscale	12 weeks [41]	Irritative subscale	NA	−11.7 (−10.9, 12.6)^c^
		Unclear [42]			-11.9
	UDI-Subscale	12 weeks [41]	Stress subscale	NA	−13.1 (−12.3, −13.9) ^c^
	Urinary Impact Questionnaire (UIQ)	12 weeks [41]	Total score	NA	−11.7 (−10.9, 12.6) ^c^
		12 weeks [43]			-15
	Overactive Bladder Questionnaire (OAB-q)	Unclear [42]	Total score	NA	4.3
	OAB-q-Subscale	Unclear [42]	Symptom severity		-7.5

*MID* Minimal important difference, *NA* Not applicable, *SUI* Stress urinary incontinence; *PROM* Patient-reported outcome measure; *UUI* Urgency urinary incontinence; *-* No quantitative estimate was provided

^a^the effect size represents the standardized change of the score at the target instrument. It can be classified in small, medium, and large effect sizes considering 0.20, 0.50, and 0.80, respectively

^b^values presented in this table are related to the MID reported in points, according to each specific PROM (questionnaire)

^c^MID (95%CI)

**Table 4:** Quantitative results from the studies included in the present systematic review, according to anchor-based methods

Nº	PROM	Anchor	Follow-up	Total score or domains	Level of improvement used during the analysis	Units	Values (MID)	Credibility (MID tool)	Certainty of evidence (GRADE)
1.1	Australian Pelvic Floor Questionnaire	Patient Global Impression of Improvement questionnaire (PGI-I)	6 weeks [49]	Bladder domain	Slight	Mean (SD)	1.5 (0.9)	Low	⨁◯◯◯ Very low
1.2				Global score			2.9 (2.9)	Low	
1.3	Incontinence Quality of Life (I-QOL)	PGI-I	14 weeks [40]	Total score	Slight	Mean (SD)	6.3 (10.2)	Low	⨁◯◯◯ Very low
			14 weeks [40]		Slight	MID	2.5		
1.4			6 weeks [39]		Slight	Points	2	Low	
1.5			6 weeks [39]		Strong	Points	13		
1.6		PGI-I	14 weeks [40]	Avoidance and Limiting Behavior	Slight	Mean (SD)	2.7 (11.6)	Low	
				Psychosocial Impacts			5.6 (10.8)		
				Social Embarrassment			6.8 (14.8)		
1.7		PGI-I	14 weeks [40]	Avoidance and Limiting Behavior	Slight	MID	2.5	Low	
				Psychosocial Impacts			2.3		
				Social Embarrassment			2.6		
1.8		Voiding diary	6 weeks [39]	Total score	Strong	Points	5	Low	
1.9		Pad test	6 weeks [39]	Total score	Strong	Points	2	Low	
1.10	International Consultation on Incontinence Questionnaire - Short Form (ICIQ-SF)	PGI-I	16 weeks [45]	Total score	Slight	MID (SD)	2.52 (2.56)	Low	⨁◯◯◯ Very low
1.11			1-year [48]	Total score	Strong	MID (95%CI)	3.8 (2.7, 4.9)	Low	
1.12			1-year [46]	Total score	Strong	MID (95%CI)	−4.8 (−5.6, −3.9)	Low	
1.13			2-years [46]	Total score	Strong	MID (95%CI)	−4.2 (−5.1, −3.4)	Low	
1.14			12 weeks: SIMS trial [50]	Total score	Slight	MID (95%CI)	–3.8 (–5.5, –2.0)	Low	
1.15			1-year: SIMS trial [50]	Total score	Slight	MID (95%CI)	–4.7 (–6.1, –3.2)	Low	
1.16			2-years: SIMS trial [50]	Total score	Slight	MID (95%CI)	–3.0 (–4.5, –1.6)	Low	
1.17			3-years: SIMS trial [50]	Total score	Slight	MID (95%CI)	–1.6(–3.0, –0.2)	Low	
1.18			16 weeks: OPAL trial [50]	Total score	Slight	MID (95%CI)	–2.0(–2.7, –1.2)	Low	
1.19			1-year: OPAL trial [50]	Total score	Slight	MID (95%CI)	–1.7(–2.5, –1.0)	Low	
1.20			2-years: OPAL trial [50]	Total score	Slight	MID (95%CI)	–1.9(–2.7, –1.1)	Low	
1.21		Satisfaction	1-year [48]	Total score	Strong	MID (95%CI)	4.4 (3.4, 5.4)	Low	
1.22			1-year [46]				-5.2 (−6.6, −3.7)	Low	
1.23			2-years [46]				−4.3 (−5.8, −2.8)	Low	
1.24		Voiding diary	1-year [48]	Total score	Strong	MID (95%CI)	3.8 (2.1, 5.5)	Low	
1.25			1-year [46]		Moderate		−4.8 (−7.5, −2.1)	Low	
					Strong		−4.5 (−5.8, −3.1)	Low	
1.26			2-years [46]		Moderate		−3.1 (−5.7, −0.5)	Low	
					Strong		−4.1 (−5.3, −3.0)	Low	
1.27		Pad-test	1-year [48]	Total score	Strong	MID (95%CI)	3.4 (2.0, 4.8)	Low	
1.28		UDI	1-year [46]	Total score	Strong	MID (95%CI)	-5.1 (−5.9, −4.2)	Low	
1.29			2-years [46]				-4.2 (−5.0, −3.4)	Low	
1.30		IIQ	1-year [46]	Total score	Strong	MID (95%CI)	-5.7 (-6.8, -4.6)	Low	
1.31			2-years [46]				-4.2 (−5.2, −3.1)	Low	
1.32	ICIQ-Lower Urinary Tract Symptoms Quality of Life (ICIQ-LUTSqol)	PGI-I	16 weeks [45]	Total score	Slight	MID (SD)	6.9 (2.9, 11.0)	Low	⨁◯◯◯ Very low
1.33			1-year [48]		Strong	MID (95%CI)	3.71 (4.95)	Low	
1.34		Satisfaction	1-year [48]	Total score	Strong	MID (95%CI)	5.4 (1.3, 9.5)	Low	
1.35		Voiding diary	1-year [48]	Total score	Strong	MID (95%CI)	4.8 (1.4, 10.9)	Low	
1.36		Pad-test	1-year [48]	Total score	Strong	MID (95%CI)	5.2(0.5, 9.8)	Low	
1.37	Urinary Impact Questionnaire (UIQ)	PGI-I	12 weeks [41]	Total score	Slight	MID (95%CI)	−6 (−22.8, 9.8)	Low	⨁◯◯◯ Very low
1.38		Satisfaction	12 weeks [43]	Total score	Slight	MID	-28	Low	
1.39		Voiding diary	12 weeks [41]	Total score	Moderate	MID (95%CI)	−17.0 (−32.9, −1.1)	Low	
1.40		Incontinence Severity Index	12 weeks [41]	Total score	Calculations were based on 1 point of difference for each level of severity that had changed (very severe to severe, severe to moderate, moderate to slight)	MID (95%CI)	−16. (−26.4, −5.7)	Low	
1.41		10-cm VAS score indicating the severity of symptoms	12 weeks [43]	Total score	Slight	MID	-14	Low	
1.42	Urogenital Distress Inventory (UDI)	PGI-I	12 weeks [41]	Total score	Slight	MID (95%CI)	−6.4 (−19.4, 6.5)	Low	
1.43		Global Perception of Improvement	10 weeks [42]	Total score	Moderate	MID (Min-Max)	-35.3 (-51.9, -18.8)	Low	⨁◯◯◯ Very low
1.44			8 months [42]				-42.5 (-56.5, -28.6)	Low	
1.45		Patient Satisfaction Questionnaire	10 weeks [42]	Total score	Strong	MID (Min-Max)	-38.1 (-63.3, -12.8)	Low	
1.46			8 months [42]				-40.5 (-56.8, -24.1)	Low	
1.47		Incontinence Severity Index	12 weeks [41]	Total score	Calculations were based on 1 point of difference for each level of severity that had changed (very severe to severe, severe to moderate, moderate to slight)	MID (95%CI)	−11.1 (−19.8, −2.3)	Low	
1.48		Satisfaction	12 weeks [43]	Total score	Slight	MID	-14	Low	
1.49		Voiding diary	12 weeks [41]	Total score	Moderate	MID (95%CI)	-22.4 (-36.5, -8.2)	Low	
1.50			10 weeks [42]			MID (Min-Max)	-41.2 (-67.6; -14.9)	Low	
1.51			8 months [42]			MID (Min-Max)	-36.2(-56.5, -15.8)	Low	
1.52		Voiding diary	8 months [42]	Total score	Strong	MID (Min-Max)	44.6 (-58.3, -30.9)	Low	
1.53		10-cm VAS score indicating the severity of symptoms	12 weeks [43]	Total score	Slight	MID	-30	Low	
1.54	UDI-Subscale	Global Perception of Improvement	10 weeks [42]	Irritative subscale	Moderate	MID (Min-Max)	-22.9 (-30.5, -15.4)	Low	⨁◯◯◯ Very low
1.55			8 months [42]				-17.9 (-23.9, -11.9)	Low	
1.56		Patient Satisfaction Questionnaire	10 weeks [42]	Irritative subscale	Strong	MID (Min-Max)	-25.3 (-37.1, -13.6)	Low	
1.57			8 months [42]				-20.1 (-27.2, -12.9)	Low	
1.58		Voiding diary	10 weeks [42]	Irritative subscale	Moderate	MID (Min-Max)	-14.5 (-26.6, -2.4)	Low	
1.59			8 months [42]				-19.1 (-28.2, -10.0)	Low	
1.60		Voiding diary	10 weeks [42]	Irritative subscale	Strong	MID (Min-Max)	-18.1 (-24.0, -12.1)	Low	
1.61			8 months [42]				-19 (-25.3, -12.8)	Low	
1.62	UDI-Subscale	PGI-I	12 weeks [41]	Stress subscale	Slight	MID (95%CI)	−4.6 (−12.7, 3.5)	Low	⨁◯◯◯ Very low
1.63		Voiding diary	12 weeks [41]	Stress subscale	Moderate	MID (95%CI)	-16.5 (−24.5, −8.3)	Low	
1.64		Incontinene Severity Index	12 weeks [41]	Stress subscale	Calculations were based on 1 point of difference for each level of severity that had changed (very severe to severe, severe to moderate, moderate to slight)	MID (95%CI)	−7.5 (−12.7, −2.3)	Low	
1.65	Overactive Bladder Questionnaire (OAB-q)	Global Perception of Improvement	10 weeks [42]	Total score	Moderate	MID (Min-Max)	-20.6 (-27.8, -13.4)	Low	⨁◯◯◯ Very low
1.66			8 months [42]				-12.7 (-18.4, -7.0)	Low	
1.67		Patient Satisfaction Questionnaire	10 weeks [42]	Total score	Strong	MID (Min-Max)	-25.0 (-36.2, -13.7)	Low	
1.68			8 months [42]				-14.9 (-22.1, -7.7)	Low	
1.69		Voiding diary	10 weeks [42]	Total score	Moderate	MID (Min-Max)	-16.3 (-27.2, -5.5)	Low	
1.70			8 months [42]				-19.3 (-27.6, -11.1)	Low	
1.71		Voiding diary	10 weeks [42]	Total score	Strong	MID (Min-Max)	-16.5 (-21.9, -11.1)	Low	
1.72			8 months [42]				-18.2 (-23.8, -12.5)	Low	
1.73	International Consultation on Incontinence Questionnaire – Female Lower Urinary Tract Symptoms (ICIQ-FLUTS)	PGI-I	1-year: SIMS trial [50]	Incontinence domain	Slight	MID (95%CI)	–1.8(–3.1, –0.6)	Low	⨁◯◯◯ Very low
1.74			2-years: SIMS trial [50]				–3.2(–4.4, –2.0)	Low	
1.75			3-years: SIMS trial [50]				–0.7(–1.8,0.5)	Low	
1.76			6 months: OPAL trial [50]				–1.4(–2.1, –0.7)	Low	
1.77			1-year: OPAL trial [50]				–1.3(–1.9, –0.6)	Low	
1.78			2-years: OPAL trial [50]				–1.9(–2.6, –1.1)	Low	

**Fig. 2:**
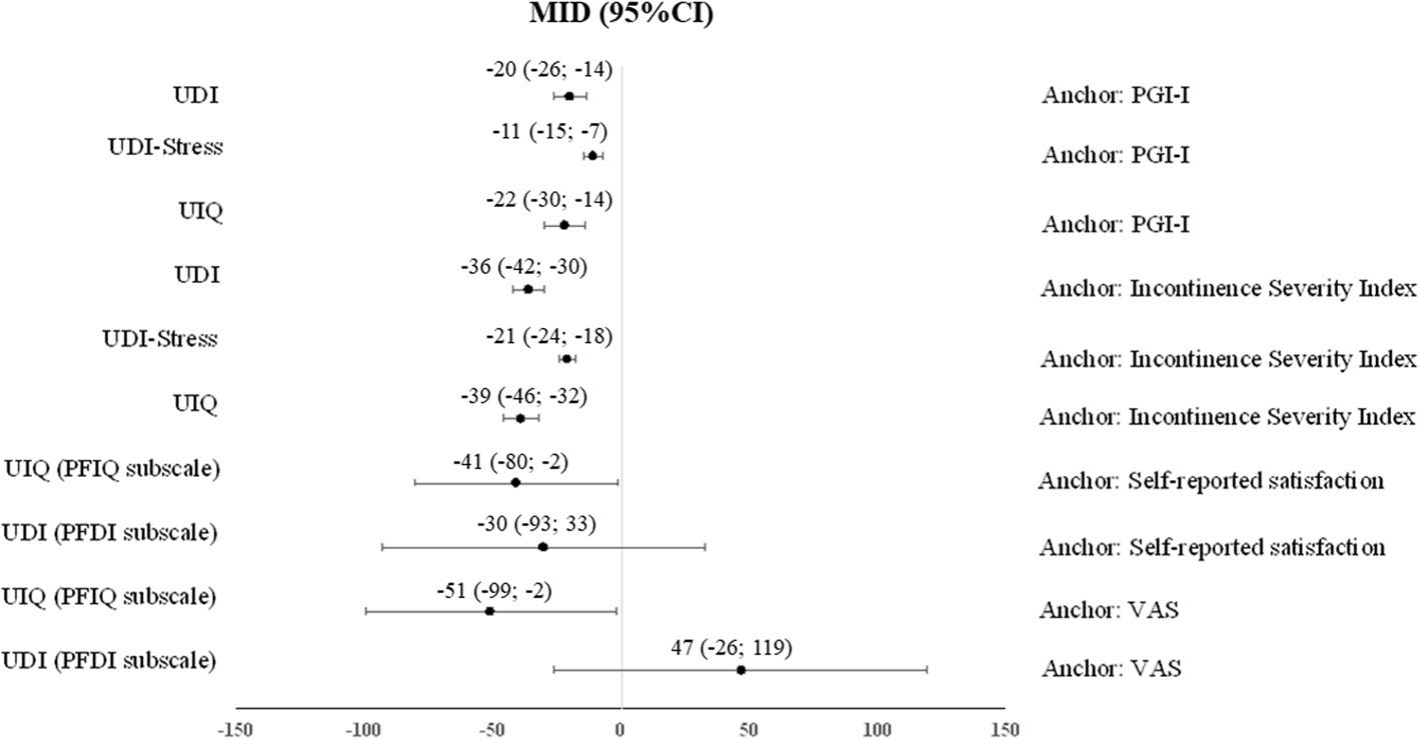
MIDs estimations and 95%CI considering the slight improvement reported by the authors, for MIDs ranging from 0 to 10 points in their respective PROMS. CI: confidence interval; ICIQ-SF: International Consultation on Incontinence Questionnaire - Short Form; I-QOL: Incontinence Quality of Life; MID: minimal important difference; PGI-I: Patient Global Impression of Improvement questionnaire

**Fig. 3:**
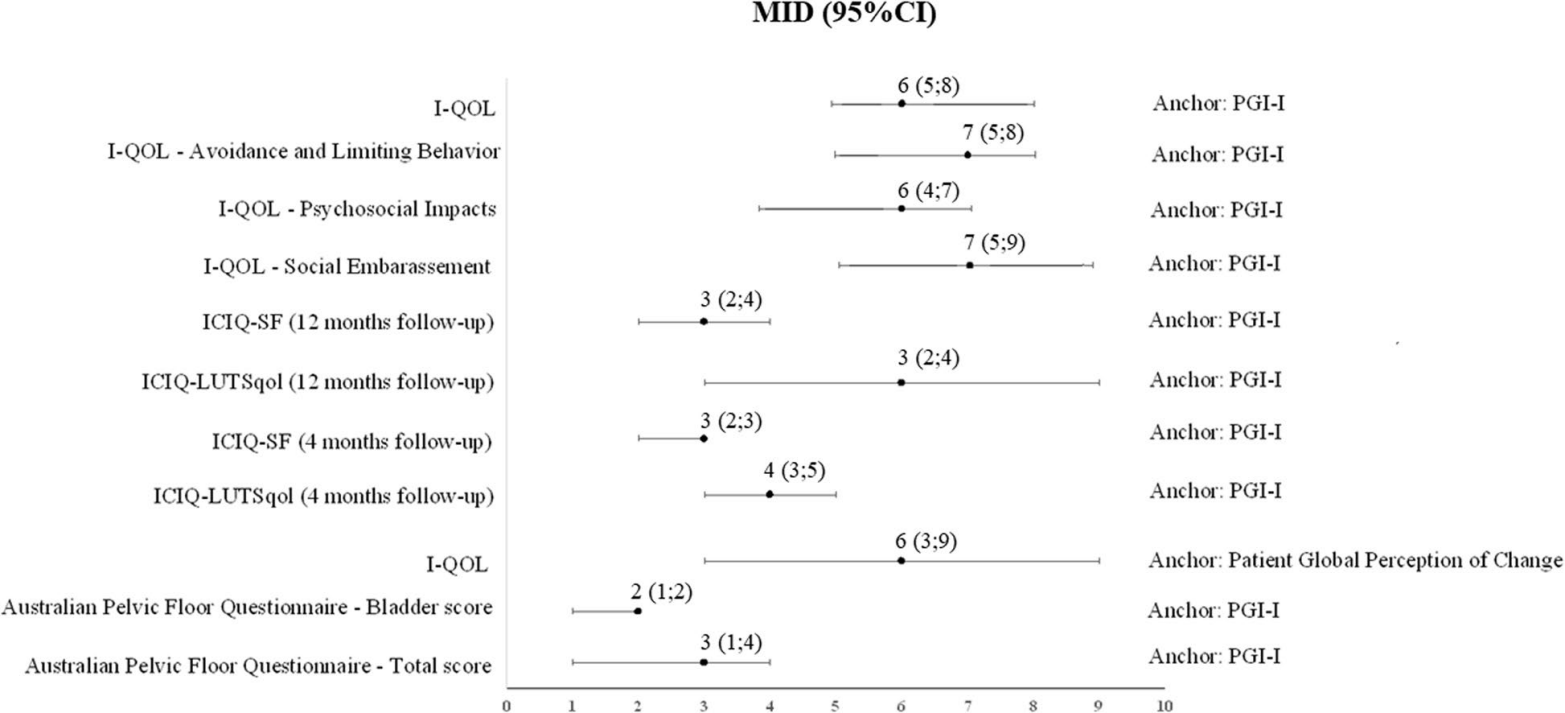
MIDs estimations and 95%CI considering a slight improvement reported by the authors, for MIDs ranging from -150 to +150 points in their respective PROMS. CI: confidence interval; MID: minimal important difference; PFDI: Pelvic Floor Distress Inventory; PFIQ: Pelvic Floor Impact questionnaire; PGI-I: Patient Global Impression of Improvement questionnaire; UDI: Urogenital Distress Inventory; UIQ: Urinary Impact Questionnaire; VAS: visual analogue scale

Figure 2 provides the minimal important difference estimates ranging from 0 to 10 points in their respective patient-reported outcome measures from included studies, considering the score of the patient-reported outcome measures related to the smallest improvement of UI. Figure 3 presents minimal important differences which had a higher range of scores in the patient-reported outcome measures (-150 to +150).

### Certainty of evidence

All the minimal important differences reported by anchor-based methods were considered with very low quality of evidence. For more details about GRADE, please check Appendix 9.

All studies [39–43, 45, 46, 48–50] presented very serious concerns about the risk of bias, which means that they presented low credibility in calculating and reporting the minimal important difference according to anchor-based methods. There was also serious and very serious inconsistency in the studies.

We downgraded the quality/certainty of the evidence for inconsistency (ICIQ-SF [45, 46, 48], ICIQ-LUTSqol [45, 48], UDI [41, 42]) and indirectness of studies that did not include in their analysis only the population with minimal improvement in their criteria (according to the minimal important difference definition and main question of the present review). Considering this last criterion, three patient-reported outcome measures presented “not serious” indirectness (Australian Pelvic Floor Questionnaire [49], IQOL-Subscores [40], UIQ [41, 43]), while four studies showed “serious” indirectness (UDI [41, 42], UDI-Irritative subscale [42], UDI-Stress subscale [41], OAB-q [42]) and three studies showed “very serious” indirectness (IQOL-Total score [39, 40], ICIQ-SF [45, 46, 48], ICIQ-LUTSqol [45, 48]).

Most parts of the outcomes included a sample size >300, although two patient-reported outcome measures were considered with a serious imprecision (UD/I-Irritative scale [42], OAB-q [42]), while one outcome was considered to have a very serious imprecision (Australian Pelvic Floor Questionnaire [49]).

Publication bias was not considered for this systematic review since the search process was comprehensive and exhaustive.

## Discussion

We included 12 studies that reported minimal important differences in outcome measures used when managing female urinary incontinence, with high variability in methods and values. The minimal important differences from thirteen different patient-reported outcome measures were reported, most of time according to anchor-based methods, using ten different anchors. However, all studies with anchor-based methods presented a low credibility and very low overall certainty. Also, minimally important differences values seem to change according to the time points that are used to generate the minimally important differences (i.e., follow-up of 4 or 6 weeks, 12 and 24 months), the characteristics of the population (i.e., type of urinary incontinence) and different anchors used.

Similar to a previous review [51], minimal important differences provided by distribution based-methods were smaller than the ones provided by anchor based-methods, which could possibly suggest that a smaller change is necessary to represent a clinically significant difference [52]. It is known that distribution based-methods only consider the distribution of the scores on their calculations and they are usually related to the variation/change that was observed in a standardized way around the mean. For this reason, previous literature suggested that anchor-based methods should be preferred over distribution-based methods [17].

A possible explanation for the wide variability around these minimal important differences may be related to the level of improvement of patients considered during data analysis. Although some authors already hypothesized that there is neither consensus nor evidence about what is the best criteria to determine the minimal important difference using anchor based-methods [17, 53], it should be pointed out that calculations that include groups of participants who considered themselves to have improved moderately or greatly after an intervention could lead to different minimal important differences estimations and it does not follow the original concept of minimal important difference that includes the “smallest difference” in scores that the individuals consider to be beneficial [54]. In the present systematic review, the majority of studies did not consider the smallest change of improvement (as perceived by the patients) in their calculations, so future studies could be biased if they consider these values in the estimation of their sample size, or even on interpreting their results. Halme et al. [55] published a study that compiled estimations for calculating sample sizes of trials to treat female urinary incontinence according to minimal important differences. In their statistical analysis, the authors included participants that reported a “very much better” improvement after treatment, which does not represent the smallest difference perceived by the patient.

Previous studies [26, 53] recognized the need of validating studies for anchors that are commonly used for data collection about the perception of patients regarding a treatment. Furthermore, there is a need for standardizing the procedures to assess important changes for the patient, by establishing a valid and specific question for that. The lack of validation a standardizing implies a variability in the results, due to the application of different anchors to calculate minimal important differences [53], generating inconsistency between studies that assess minimal important differences.

The literature suggests that anchors should be selected based on it´s relevance and should lay proximal to the construct assessed by the patient-reported outcome measures, which is usually analyzed by the correlation between the tools (anchor and patient-reported outcome measures). Also, researchers and clinicals should consider the characteristics of the sample and severity of the disease in order to define the adequate anchor. In addition, this rationale should be based on previous guidance and scientific evidence [29]. A previous study also found that derived minimal important differences are highly variable due to the discrepancy in study designs, methods, and concepts used when calculating the minimal important differences [26]. These results agree with the present review.

The newly developed tool used to assess the credibility of the derived minimal important differences according to anchor-based methods showed that the studies presented low credibility. Most studies did not report a pre-requisite of minimal important differences calculation, which is the correlation between the patient-reported outcome measures and the anchor. In addition, only three studies [42, 43, 46] reported the correlations between anchors and patient-reported outcome measure scores during follow-up. This missing information could also help to explain the variability found from the minimal important difference values [53]. Considering that anchor and patient-reported outcome measures should be measured in the same or similar underlying constructs, correlations between tools show that both tools are closely linked. Therefore, anchors with absence or low correlation will provide inaccurate minimal important difference estimates [34].

Attention should be drawn to methodological issues related to the calculations and reports of minimally important differences while interpreting the results reported by the literature. It is important to evaluate the credibility of minimal important difference since there is a substantial misunderstanding of methods and concepts that can lead to incorrect reporting of minimal important difference values. Authors should follow some guidance while conducting studies with this aim. This information could be found in previous studies [17] and also by interpreting and incorporating the items assessed by the credibility tool [34] in future studies.

This review contributes substantially to Women’s Health research. A summary of the minimal important differences for outcomes related to urinary symptoms in the literature may contribute to evidence-based practice, by complementing statistical results with clinicians’ clinical experience and patients’ perception of a treatment [17, 28]. It may result in a new direction for the treatment of urinary symptoms since it brings a focus to interventions that are clinically relevant and can be successfully implemented in clinical practice. Moreover, a new interpretation of results from the literature may be incorporated, as we bring to focus the estimates that might be used to classify results from studies as clinically relevant, not only with statistical power. It may highlight in previous studies that an over- or underestimation could possibly have occurred in the past by interpreting only results from statistical analysis. In addition, our results could facilitate the design and planning of future studies such as generating accurate sample size calculations, determining best outcome measures, and therefore, facilitating the future update of clinical research into practice. Therefore, researchers are encouraged to incorporate these outcomes in their clinical studies to measure the effectiveness of interventions, taking into consideration not only statistical significance but also clinical relevance.

This systematic review followed a rigorously methodological sequence which included the preparation and registration of a protocol for the review, and a systematic search of the most important databases. The eligibility, data extraction, and credibility of the studies were performed by two independent researchers. Moreover, the present review only included studies that reported minimal important differences according to analysis that are already recommended by previous guidelines. We reported which tools already have a minimal important difference that is available to be used in clinical research. In addition, we synthesized the steps and information that are necessary to calculate and analyze the minimal important difference, besides the guidance to help researchers to interpret it correctly. Furthermore, some limitations and misconceptions related to minimal important differences raised from the results of the present review were emphasized.

The present systematic review has some limitations. The limited number of studies included did not allow us to perform sub-analysis according to the type of urinary incontinence, methods of calculation (i.e., distribution or anchor-based method), and/or anchors used during data analysis. Moreover, it was not possible to assess the credibility of studies that reported minimal important differences according to distribution-based methods, as the tool described by Devji et al. [34] was developed to evaluate studies that reported minimal important differences by anchor-based methods (which is the most accepted method to generate minimal important differences). In addition, although guidance exists on how to apply the tool, some clarity was needed on some specific points, especially when deriving a final assessment. Authors from the present review agreed on decision rules to assess the credibility of the minimally important differences derived in the analyzed studies. These decision rules might be considered arbitrary; however, they were based on similar decision rules done in the context of RoB assessment of RCTs.

Although we provide minimal important differences derived by anchor based-methods according to the smallest improvement based on the mean change, our analysis was restricted to the availability of data reported by the studies, such as the scores of patient-reported outcome measures of the group of patients who considered themselves “a little better”. In cases where data was not available, the calculation was not possible, which limited the information reported in our review.

We planned to triangulate minimal important differences derived from the same patient-reported outcome measures, considering the method of calculation (i.e., distribution or anchor based-method) and/or anchors used during data analysis. However, regarding the variability among the studies, it was not possible to calculate one single value of minimal important difference for each patient-reported outcome measure. This is a common limitation among systematic reviews that try to compile minimal important differences available for different patient-reported outcome measures [26, 56]. Previous reports^39,58,64,6^ concluded that minimal important differences could not be interpreted as a constant characteristic and a universally empirical score could not be derived. Instead, it is recommended that minimal important difference is analyzed and considered according to the severity of the condition during the baseline, the type of treatment, the units of the patient-reported outcome measures, the conditions of the population, and the context where the patient is located [29, 51, 56, 57]. In addition, it seems that minimal important differences can also change according to the different characteristics of the population [53]. That was also the case in the present study, as it was also possible to notice that minimal important differences from a population with urgency urinary incontinence [42] were different for the same patient-reported outcome measures in a sample with stress urinary incontinence [41]. Therefore, authors should be aware to include these characteristics in their reports about minimal important differences.

Moreover, our study did not explore the factors that could lead to the variability among minimal important differences reported by the authors through sensitivity analysis due to the limited number of studies. Future studies should perform specific statistical analysis to identify which are the factors that could be associated with this variability in order to reduce the disparity and variability among studies. In addition, future studies should be aware of the recommendations regarding the reports that include minimal important differences and should report: 1) the scores from the baseline and follow-up, in order to enable future explorations, even considering the variability among studies [26]; 2) improve the reports regarding the correlations found between anchors and patient-reported outcome measures, during baseline and follow-up; 3) conduct studies that aim to validate anchors often used in studies of Women’s Health.

Twelve different patient-reported outcome measures with respective minimal important differences for outcomes related to urinary incontinence were found in the literature, considering 48 and 65 minimal important differences reported according to distribution- and anchor-based methods, respectively. Values based on distribution-based methods were smaller than the anchor-based method. However, the credibility and certainty of evidence of all the minimal important differences related to urinary incontinence measures reported by anchor-based methods were low and very low. The methodology to derive minimal important difference for outcomes related to urinary incontinence need to be improved.

### Supplementary Information

**Supplementary Material 1.** 

**Supplementary Material 2.** 

**Supplementary Material 3.** 

**Supplementary Material 4.** 

**Supplementary Material 5.** 

**Supplementary Material 6.** 

**Supplementary Material 7.**

**Supplementary Material 8.** 

**Supplementary Material 9.** 

## Data Availability

Not applicable.
