# Supplementary material for: The minimal important difference of patient-reported outcome measures related to female urinary incontinence: a systematic review
Source: BMC Med Res Methodol. 2024 Mar 8;24:60. doi: 10.1186/s12874-024-02188-4 (PMC10921720; doi:10.1186/s12874-024-02188-4)
Supplement: Supplementary file 1 — Supplementary Material 1. [file 12874_2024_2188_MOESM1_ESM.docx]

**Appendix 1**. Distribution- and anchor-based methods available to calculate MID according to Armijo-Olivo et al^18^.

| **Methods to calculate** | **Definition/Interpretation** |
| --- | --- |
| **Distribution-based methods** | |
| Effect Size (ES) | The standardize change of the score at the target instrument.  The ES have been considered as small, moderate, and large according to 0.2, 0.5, and 0.8, respectively.  Recently, new values were proposed: small, medium, and large effect sizes were 0.14, 0.31, and 0.61, respectively. |
| Standardized Response Mean (SRM) | Similar to the ES.  The difference between ES is that the formula to calculate the SRM uses the SD of the change instead of the pooled SD. |
| Standard Error of Measurement (SEM) | The random measurement error, quantifying the variability.  To be considered as a true change, the value should be above the baseline SEM.  Changes smaller than this amount cannot be distinguish from variability due to measurement error.  The estimate can use 1 or 1.96, 2, or 2.77 of SEM, considering small (1 of SEM), and moderate (1.96, 2, or 2.77 of SEM), respectively. |
| Standard Deviation (SD) | The standard deviation of the scores.  The most common multiple of SD used was 0.5*SD. However, it is possible to calculate MID according 0.3SD and also 0.2SD.  Previous studies associated the multiples correspond to small (0.2), medium (0.5), or large (0.8) effects. |
| Minimal Detectable Change (MDC) | MDC can indicate whether 10% (MDC_90_) or 5% (MDC_95_) of patients who truly are unchanged will display fluctuations greater than the calculated threshold or MDC. |
| **Anchor-based methods** | |
| Change Difference (CD) | The CD can be defined as the difference obtained in the outcome of interest (target instrument) between patients who improved and those who did not improve.  It can be useful to establish a cut-off based on values from patients that showed small, moderate, or large changes, according to an anchor-based method. |
| Receiver Operating Curve (ROC) | A type of statistical analysis that uses discriminative tests to calculate the cut-off value, which can differentiate individuals who improved from the ones that did not improve or got worse.  By the area under the curve (AUC) analysis, it is possible to measures the accuracy of the target instrument to classify patients and the responsiveness of the instrument. |
| Regression analysis (REG) | There are two available regression analysis: linear and logistic regression.  The MID is considered as the β coefficient estimate of the anchor, for the regression of the measure score or change in the anchor regressor.  In linear regression analysis, each one point of increase in the dependent variable, will increase the β value (MID) of the patients evaluated.  In logistic regression, each point that increases in the target scale, will improve the likelihood of the anchor in the odds ratio value. |
| Average Change (AC) | Defined as the average of the score change or mean difference (MID) observed by the patient who are classified as responders according to the cut-off point from the anchor. |
| Minimal Detectable Change (MDC) | Upper limit of the 95% confidence interval (MID) of the average change in non-responders (according to the anchor´s cut-off point). |
| Equipercentile linking (EL) | This test identifies the scores on the anchor and the target instrument which represents the same percentile rank. |

Source: Adapted from Armijo-Olivo et al., 2021^18^.
