# Supplementary material for: The minimal important difference of patient-reported outcome measures related to female urinary incontinence: a systematic review
Source: BMC Med Res Methodol. 2024 Mar 8;24:60. doi: 10.1186/s12874-024-02188-4 (PMC10921720; doi:10.1186/s12874-024-02188-4)
Supplement: Supplementary file 2 — Supplementary Material 2. [file 12874_2024_2188_MOESM2_ESM.docx]

**Appendix 2**. Search strategy with the key-words used during database searchers.

| Database | Search terms |
| --- | --- |
| Ovid MEDLINE(R) In-Process & Other Non-Indexed Citations, Ovid MEDLINE(R) Daily and Ovid MEDLINE(R)  1946 to Present | 1. urinary incontinence/ or urinary incontinence, stress/ or urinary  2. (urinary incontinence or stress urinary incontinence or urgency urinary incontinence or urge-incontinence or mixed urinary incontinence or urinary symptoms or urinary lost or urine losses or lower urinary tract symptoms or bladder symptoms or overactive bladder or stress incontinence or mixed incontinence or functional incontinence).mp. [mp=title, abstract, heading word, drug trade name, original title, device manufacturer, drug manufacturer, device trade name, keyword, floating subheading word, candidate term word]  3. 1 or 2  4. (kings health questionnaire or overactive bladder-validated 8-question awareness tool or international consultation on incontinence questionnaire or iciq or iciq-ui sf).mp. [mp=title, abstract, heading word, drug trade name, original title, device manufacturer, drug manufacturer, device trade name, keyword, floating subheading word, candidate term word]  5. (((kings health questionnaire or overactive bladder-validated 8-question awareness tool or international consultation on incontinence questionnaire or iciq or iciq-ui sf or pelvic floor distress inventory or pfdi or pelvic floor impact questionnaire or pfiq or bladder diary or pad-test or medical outcomes study 36-item short-form health survey or bristol female lower urinary tract symptoms or incontinence impact questionnaire or incontinence severity index or urogenital distress inventory or urinary incontinence-specific quality of life instrument or international consultation on incontinence modular questionnaire or iciq-fluts or pelvic organ prolapse urinary incontinence sexual questionnaire or urgency perception scale or urinary incontinence severity score or incontinence outcome questionnaire or women irritative prostate symptoms score or pad weighing or wet checks or overactive bladder questionnaire or stress) and urge incontinence and quality of life questionnaire) or voiding diary or bladder diary or urinary diary or iciq bladder diary).mp. [mp=title, abstract, heading word, drug trade name, original title, device manufacturer, drug manufacturer, device trade name, keyword, floating subheading word, candidate term word]  6. (stress test or cough stress test or urodynamics or symptom-related questionnaires or incontinence severity index or three-level euroqol five-dimensional questionnaire or whoqol-100 or whoqol brief or 12-item short form health survey or health utilities index 3 or incontinence stress questionnaire for patients or incontinence quality of life index or urgent micturition or urge impact sale or urge incontinence impact questionnaire or symptom impact index for stress incontinence or urge-urinary distress inventory or quality of life in persons with urinary incontinence or ics quality of life or contilife or urinary incontinence severity score or urinary incontinence handicap inventory or york incontinence perceptions scale or vaginal palpation or digital palpation).mp. [mp=title, abstract, heading word, drug trade name, original title, device manufacturer, drug manufacturer, device trade name, keyword, floating subheading word, candidate term word]  7. (stress test or cough stress test or urodynamics or symptom-related questionnaires or incontinence severity index or three-level euroqol five-dimensional questionnaire or whoqol-100 or whoqol brief or 12-item short form health survey or health utilities index 3 or incontinence stress questionnaire for patients or incontinence quality of life index or urgent micturition or urge impact sale or urge incontinence impact questionnaire or symptom impact index for stress incontinence or urge-urinary distress inventory or quality of life in persons with urinary incontinence or ics quality of life or contilife or urinary incontinence severity score or urinary incontinence handicap inventory or york incontinence perceptions scale or vaginal palpation or digital palpation).mp. [mp=title, abstract, heading word, drug trade name, original title, device manufacturer, drug manufacturer, device trade name, keyword, floating subheading word, candidate term word]  8. minimal important difference.mp.  9. exp *minimal clinically important difference/  10. (clinical* important difference? or clinical* meaningful difference? or clinical* meaningful improvement? or clinical* relevant mean difference? or clinical* significant change? or clinical* significant difference? or clinical* important improvement? or clinical* meaningful change? or mcid or minim* clinical* important or minim* clinical* detectable or minim* clinical* significant or minim* detectable difference? or minim* important change? or minim* important difference? or smallest real difference? or subjectively significant difference?).tw.  11. (clinical relevance or clinical relevance or clinically relevant or clinical relevant or clinical significance or clinically significance or clinically important difference or clinical important difference or clinically significant or clinically significant improvement or clinical significant improvement or clinically meaningful improvement or clinical meaningful improvement or clinically relevant improvement or clinical relevant improvement or clinically meaningful or clinical meaningful or meaningful improvement or clinical significant difference or clinically significant difference or clinical important improvement or clinically important improvement or clinical meaningful change or clinically meaningful change or mcid or minimally clinical important or minimally clinically important or minimum clinical important or minimum clinically important or minimal clinical important or minimal clinically important or minimum clinical detectable or minimum clinically detectable or minimal clinical detectable or minimal clinically detectable or minimum clinical significant or minimum clinically significant or minimal clinical significant or minimal clinically significant or minimum detectable difference or minimum detectable difference or minimal detectable difference or minimal detectable difference or minimum important change or minimum important change or minimal important change or minimal important change or minimum important difference or minimum important difference or minimal important difference or minimal important difference or smallest real difference or subjectively significant difference).mp. [mp=title, abstract, heading word, drug trade name, original title, device manufacturer, drug manufacturer, device trade name, keyword, floating subheading word, candidate term word]  12. 4 or 5 or 6 or 7  13. 8 or 9 or 10 or 11  14. 3 and 12 and 13  15. exp urine incontinence/ or urinary incontinency.mp. or exp stress incontinence/ or exp incontinence/  16. I or 2 or 15  17. 12 and 13 and 16 |
| Embase (Ovid) 1974 to 2017 Week 23 | 1. urinary incontinence/ or urinary incontinence, stress/ or urinary  2. (urinary incontinence or stress urinary incontinence or urgency urinary incontinence or urge-incontinence or mixed urinary incontinence or urinary symptoms or urinary lost or urine losses or lower urinary tract symptoms or bladder symptoms or overactive bladder or stress incontinence or mixed incontinence or functional incontinence).mp. [mp=title, abstract, heading word, drug trade name, original title, device manufacturer, drug manufacturer, device trade name, keyword, floating subheading word, candidate term word]  3. 1 or 2  4. (kings health questionnaire or overactive bladder-validated 8-question awareness tool or international consultation on incontinence questionnaire or iciq or iciq-ui sf).mp. [mp=title, abstract, heading word, drug trade name, original title, device manufacturer, drug manufacturer, device trade name, keyword, floating subheading word, candidate term word]  5. (((kings health questionnaire or overactive bladder-validated 8-question awareness tool or international consultation on incontinence questionnaire or iciq or iciq-ui sf or pelvic floor distress inventory or pfdi or pelvic floor impact questionnaire or pfiq or bladder diary or pad-test or medical outcomes study 36-item short-form health survey or bristol female lower urinary tract symptoms or incontinence impact questionnaire or incontinence severity index or urogenital distress inventory or urinary incontinence-specific quality of life instrument or international consultation on incontinence modular questionnaire or iciq-fluts or pelvic organ prolapse urinary incontinence sexual questionnaire or urgency perception scale or urinary incontinence severity score or incontinence outcome questionnaire or women irritative prostate symptoms score or pad weighing or wet checks or overactive bladder questionnaire or stress) and urge incontinence and quality of life questionnaire) or voiding diary or bladder diary or urinary diary or iciq bladder diary).mp. [mp=title, abstract, heading word, drug trade name, original title, device manufacturer, drug manufacturer, device trade name, keyword, floating subheading word, candidate term word]  6. (stress test or cough stress test or urodynamics or symptom-related questionnaires or incontinence severity index or three-level euroqol five-dimensional questionnaire or whoqol-100 or whoqol brief or 12-item short form health survey or health utilities index 3 or incontinence stress questionnaire for patients or incontinence quality of life index or urgent micturition or urge impact sale or urge incontinence impact questionnaire or symptom impact index for stress incontinence or urge-urinary distress inventory or quality of life in persons with urinary incontinence or ics quality of life or contilife or urinary incontinence severity score or urinary incontinence handicap inventory or york incontinence perceptions scale or vaginal palpation or digital palpation).mp. [mp=title, abstract, heading word, drug trade name, original title, device manufacturer, drug manufacturer, device trade name, keyword, floating subheading word, candidate term word]  7. (stress test or cough stress test or urodynamics or symptom-related questionnaires or incontinence severity index or three-level euroqol five-dimensional questionnaire or whoqol-100 or whoqol brief or 12-item short form health survey or health utilities index 3 or incontinence stress questionnaire for patients or incontinence quality of life index or urgent micturition or urge impact sale or urge incontinence impact questionnaire or symptom impact index for stress incontinence or urge-urinary distress inventory or quality of life in persons with urinary incontinence or ics quality of life or contilife or urinary incontinence severity score or urinary incontinence handicap inventory or york incontinence perceptions scale or vaginal palpation or digital palpation).mp. [mp=title, abstract, heading word, drug trade name, original title, device manufacturer, drug manufacturer, device trade name, keyword, floating subheading word, candidate term word]  8. minimal important difference.mp.  9. exp *minimal clinically important difference/  10. (clinical* important difference? or clinical* meaningful difference? or clinical* meaningful improvement? or clinical* relevant mean difference? or clinical* significant change? or clinical* significant difference? or clinical* important improvement? or clinical* meaningful change? or mcid or minim* clinical* important or minim* clinical* detectable or minim* clinical* significant or minim* detectable difference? or minim* important change? or minim* important difference? or smallest real difference? or subjectively significant difference?).tw.  11. (clinical relevance or clinical relevance or clinically relevant or clinical relevant or clinical significance or clinically significance or clinically important difference or clinical important difference or clinically significant or clinically significant improvement or clinical significant improvement or clinically meaningful improvement or clinical meaningful improvement or clinically relevant improvement or clinical relevant improvement or clinically meaningful or clinical meaningful or meaningful improvement or clinical significant difference or clinically significant difference or clinical important improvement or clinically important improvement or clinical meaningful change or clinically meaningful change or mcid or minimally clinical important or minimally clinically important or minimum clinical important or minimum clinically important or minimal clinical important or minimal clinically important or minimum clinical detectable or minimum clinically detectable or minimal clinical detectable or minimal clinically detectable or minimum clinical significant or minimum clinically significant or minimal clinical significant or minimal clinically significant or minimum detectable difference or minimum detectable difference or minimal detectable difference or minimal detectable difference or minimum important change or minimum important change or minimal important change or minimal important change or minimum important difference or minimum important difference or minimal important difference or minimal important difference or smallest real difference or subjectively significant difference).mp. [mp=title, abstract, heading word, drug trade name, original title, device manufacturer, drug manufacturer, device trade name, keyword, floating subheading word, candidate term word]  12. 4 or 5 or 6 or 7  13. 8 or 9 or 10 or 11  14. 3 and 12 and 13  15. exp urine incontinence/ or urinary incontinency.mp. or exp stress incontinence/ or exp incontinence/  16. I or 2 or 15  17. 12 and 13 and 16 |
| CINAHL Plus with Full Text (EBSCO) 1937 to present | S1.TI (urinary incontinence or urinary)  S2. TI (urinary incontinence or stress urinary incontinence or urgency urinary incontinence or urge-incontinence or mixed urinary incontinence or urinary symptoms or urinary lost or urine losses or lower urinary tract symptoms or bladder symptoms or overactive bladder or stress incontinence or mixed incontinence or functional incontinence)  S3. S1 or S2  S4. TI (kings health questionnaire or overactive bladder-validated 8-question awareness tool or international consultation on incontinence questionnaire or iciq or iciq-ui sf)  S5. TI (((kings health questionnaire or overactive bladder-validated 8-question awareness tool or international consultation on incontinence questionnaire or iciq or iciq-ui sf or pelvic floor distress inventory or pfdi or pelvic floor impact questionnaire or pfiq or bladder diary or pad-test or medical outcomes study 36-item short-form health survey or bristol female lower urinary tract symptoms or incontinence impact questionnaire or incontinence severity index or urogenital distress inventory or urinary incontinence-specific quality of life instrument or international consultation on incontinence modular questionnaire or iciq-fluts or pelvic organ prolapse urinary incontinence sexual questionnaire or urgency perception scale or urinary incontinence severity score or incontinence outcome questionnaire or women irritative prostate symptoms score or pad weighing or wet checks or overactive bladder questionnaire or stress) and urge incontinence and quality of life questionnaire) or voiding diary or bladder diary or urinary diary or iciq bladder diary)  S6. TI (stress test or cough stress test or urodynamics or symptom-related questionnaires or incontinence severity index or three-level euroqol five-dimensional questionnaire or whoqol-100 or whoqol brief or 12-item short form health survey or health utilities index 3 or incontinence stress questionnaire for patients or incontinence quality of life index or urgent micturition or urge impact sale or urge incontinence impact questionnaire or symptom impact index for stress incontinence or urge-urinary distress inventory or quality of life in persons with urinary incontinence or ics quality of life or contilife or urinary incontinence severity score or urinary incontinence handicap inventory or york incontinence perceptions scale or vaginal palpation or digital palpation)  S7. TI (stress test or cough stress test or urodynamics or symptom-related questionnaires or incontinence severity index or three-level euroqol five-dimensional questionnaire or whoqol-100 or whoqol brief or 12-item short form health survey or health utilities index 3 or incontinence stress questionnaire for patients or incontinence quality of life index or urgent micturition or urge impact sale or urge incontinence impact questionnaire or symptom impact index for stress incontinence or urge-urinary distress inventory or quality of life in persons with urinary incontinence or ics quality of life or contilife or urinary incontinence severity score or urinary incontinence handicap inventory or york incontinence perceptions scale or vaginal palpation or digital palpation)  S8. TI (minimal important difference)  S9. TI (clinical* important difference? or clinical* meaningful difference? or clinical* meaningful improvement? or clinical* relevant mean difference? or clinical* significant change? or clinical* significant difference? or clinical* important improvement? or clinical* meaningful change? or mcid or minim* clinical* important or minim* clinical* detectable or minim* clinical* significant or minim* detectable difference? or minim* important change? or minim* important difference? or smallest real difference? or subjectively significant difference?).  S10. TI (clinical relevance or clinical relevance or clinically relevant or clinical relevant or clinical significance or clinically significance or clinically important difference or clinical important difference or clinically significant or clinically significant improvement or clinical significant improvement or clinically meaningful improvement or clinical meaningful improvement or clinically relevant improvement or clinical relevant improvement or clinically meaningful or clinical meaningful or meaningful improvement or clinical significant difference or clinically significant difference or clinical important improvement or clinically important improvement or clinical meaningful change or clinically meaningful change or mcid or minimally clinical important or minimally clinically important or minimum clinical important or minimum clinically important or minimal clinical important or minimal clinically important or minimum clinical detectable or minimum clinically detectable or minimal clinical detectable or minimal clinically detectable or minimum clinical significant or minimum clinically significant or minimal clinical significant or minimal clinically significant or minimum detectable difference or minimum detectable difference or minimal detectable difference or minimal detectable difference or minimum important change or minimum important change or minimal important change or minimal important change or minimum important difference or minimum important difference or minimal important difference or minimal important difference or smallest real difference or subjectively significant difference)  S11. S4 OR S5 OR S6 OR S7  S12. S8 OR S9 OR S10  S13. S3 AND S11 AND S12 |
| Web of Science | 1.TS=(urinary incontinence OR urinary)  2. TS=(urinary incontinence OR stress urinary incontinence OR urgency urinary incontinence OR urge-incontinence OR mixed urinary incontinence OR urinary symptoms OR urinary lost OR urine losses OR lower urinary tract symptoms OR bladder symptoms OR overactive bladder OR stress incontinence OR mixed incontinence OR functional incontinence)  3. #1 OR #2  4. TS=(kings health questionnaire OR overactive bladder-validated 8-question awareness tool OR international consultation on incontinence questionnaire OR iciq OR iciq-ui sf)  5. TS=(((kings health questionnaire OR overactive bladder-validated 8-question awareness tool OR international consultation on incontinence questionnaire OR iciq OR iciq-ui sf OR pelvic floor distress inventory OR pfdi OR pelvic floor impact questionnaire OR pfiq OR bladder diary OR pad-test OR medical outcomes study 36-item short-form health survey OR bristol female lower urinary tract symptoms OR incontinence impact questionnaire OR incontinence severity index OR urogenital distress inventory OR urinary incontinence-specific quality of life instrument OR international consultation on incontinence modular questionnaire OR iciq-fluts OR pelvic organ prolapse urinary incontinence sexual questionnaire OR urgency perception scale OR urinary incontinence severity score OR incontinence outcome questionnaire OR women irritative prostate symptoms score OR pad weighing OR wet checks OR overactive bladder questionnaire OR stress) and urge incontinence and quality of life questionnaire) OR voiding diary OR bladder diary OR urinary diary OR iciq bladder diary)  6. TS=(stress test OR cough stress test OR urodynamics OR symptom-related questionnaires OR incontinence severity index OR three-level euroqol five-dimensional questionnaire OR whoqol-100 OR whoqol brief OR 12-item short form health survey OR health utilities index 3 OR incontinence stress questionnaire for patients OR incontinence quality of life index OR urgent micturition OR urge impact sale OR urge incontinence impact questionnaire OR symptom impact index for stress incontinence OR urge-urinary distress inventory OR quality of life in persons with urinary incontinence OR ics quality of life OR contilife OR urinary incontinence severity score OR urinary incontinence handicap inventory OR york incontinence perceptions scale OR vaginal palpation OR digital palpation)  7. TS=(stress test OR cough stress test OR urodynamics OR symptom-related questionnaires OR incontinence severity index OR three-level euroqol five-dimensional questionnaire OR whoqol-100 OR whoqol brief OR 12-item short form health survey OR health utilities index 3 OR incontinence stress questionnaire for patients OR incontinence quality of life index OR urgent micturition OR urge impact sale OR urge incontinence impact questionnaire OR symptom impact index for stress incontinence OR urge-urinary distress inventory OR quality of life in persons with urinary incontinence OR ics quality of life OR contilife OR urinary incontinence severity score OR urinary incontinence handicap inventory OR york incontinence perceptions scale OR vaginal palpation OR digital palpation)  8. TS=(minimal important difference)  9. TS=(clinical* important difference? OR clinical* meaningful difference? OR clinical* meaningful improvement? OR clinical* relevant mean difference? OR clinical* significant change? OR clinical* significant difference? OR clinical* important improvement? OR clinical* meaningful change? OR mcid OR minim* clinical* important OR minim* clinical* detectable OR minim* clinical* significant OR minim* detectable difference? OR minim* important change? OR minim* important difference? OR smallest real difference? OR subjectively significant difference?).  10. TS=(clinical relevance OR clinical relevance OR clinically relevant OR clinical relevant OR clinical significance OR clinically significance OR clinically important difference OR clinical important difference OR clinically significant OR clinically significant improvement OR clinical significant improvement OR clinically meaningful improvement OR clinical meaningful improvement OR clinically relevant improvement OR clinical relevant improvement OR clinically meaningful OR clinical meaningful OR meaningful improvement OR clinical significant difference OR clinically significant difference OR clinical important improvement OR clinically important improvement OR clinical meaningful change OR clinically meaningful change OR mcid OR minimally clinical important OR minimally clinically important OR minimum clinical important OR minimum clinically important OR minimal clinical important OR minimal clinically important OR minimum clinical detectable OR minimum clinically detectable OR minimal clinical detectable OR minimal clinically detectable OR minimum clinical significant OR minimum clinically significant OR minimal clinical significant OR minimal clinically significant OR minimum detectable difference OR minimum detectable difference OR minimal detectable difference OR minimal detectable difference OR minimum important change OR minimum important change OR minimal important change OR minimal important change OR minimum important difference OR minimum important difference OR minimal important difference OR minimal important difference OR smallest real difference OR subjectively significant difference)  11. #4 OR #5 OR #6 OR #7  12. #8 OR #9 OR #10  13. #3 AND #11 AND #12 |
| Scopus | 1.TITLE-ABS-KEY(urinary incontinence OR urinary)  2. TITLE-ABS-KEY(urinary incontinence OR stress urinary incontinence OR urgency urinary incontinence OR urge-incontinence OR mixed urinary incontinence OR urinary symptoms OR urinary lost OR urine losses OR lower urinary tract symptoms OR bladder symptoms OR overactive bladder OR stress incontinence OR mixed incontinence OR functional incontinence)  3. #1 OR #2  4. TITLE-ABS-KEY(kings health questionnaire OR overactive bladder-validated 8-question awareness tool OR international consultation on incontinence questionnaire OR iciq OR iciq-ui sf)  5. TITLE-ABS-KEY(((kings health questionnaire OR overactive bladder-validated 8-question awareness tool OR international consultation on incontinence questionnaire OR iciq OR iciq-ui sf OR pelvic floor distress inventory OR pfdi OR pelvic floor impact questionnaire OR pfiq OR bladder diary OR pad-test OR medical outcomes study 36-item short-form health survey OR bristol female lower urinary tract symptoms OR incontinence impact questionnaire OR incontinence severity index OR urogenital distress inventory OR urinary incontinence-specific quality of life instrument OR international consultation on incontinence modular questionnaire OR iciq-fluts OR pelvic organ prolapse urinary incontinence sexual questionnaire OR urgency perception scale OR urinary incontinence severity score OR incontinence outcome questionnaire OR women irritative prostate symptoms score OR pad weighing OR wet checks OR overactive bladder questionnaire OR stress) and urge incontinence and quality of life questionnaire) OR voiding diary OR bladder diary OR urinary diary OR iciq bladder diary)  6. TITLE-ABS-KEY(stress test OR cough stress test OR urodynamics OR symptom-related questionnaires OR incontinence severity index OR three-level euroqol five-dimensional questionnaire OR whoqol-100 OR whoqol brief OR 12-item short form health survey OR health utilities index 3 OR incontinence stress questionnaire for patients OR incontinence quality of life index OR urgent micturition OR urge impact sale OR urge incontinence impact questionnaire OR symptom impact index for stress incontinence OR urge-urinary distress inventory OR quality of life in persons with urinary incontinence OR ics quality of life OR contilife OR urinary incontinence severity score OR urinary incontinence handicap inventory OR york incontinence perceptions scale OR vaginal palpation OR digital palpation)  7. TITLE-ABS-KEY(stress test OR cough stress test OR urodynamics OR symptom-related questionnaires OR incontinence severity index OR three-level euroqol five-dimensional questionnaire OR whoqol-100 OR whoqol brief OR 12-item short form health survey OR health utilities index 3 OR incontinence stress questionnaire for patients OR incontinence quality of life index OR urgent micturition OR urge impact sale OR urge incontinence impact questionnaire OR symptom impact index for stress incontinence OR urge-urinary distress inventory OR quality of life in persons with urinary incontinence OR ics quality of life OR contilife OR urinary incontinence severity score OR urinary incontinence handicap inventory OR york incontinence perceptions scale OR vaginal palpation OR digital palpation)  8. TITLE-ABS-KEY(minimal important difference)  9. TITLE-ABS-KEY(clinical* important difference? OR clinical* meaningful difference? OR clinical* meaningful improvement? OR clinical* relevant mean difference? OR clinical* significant change? OR clinical* significant difference? OR clinical* important improvement? OR clinical* meaningful change? OR mcid OR minim* clinical* important OR minim* clinical* detectable OR minim* clinical* significant OR minim* detectable difference? OR minim* important change? OR minim* important difference? OR smallest real difference? OR subjectively significant difference?).  10. TITLE-ABS-KEY(clinical relevance OR clinical relevance OR clinically relevant OR clinical relevant OR clinical significance OR clinically significance OR clinically important difference OR clinical important difference OR clinically significant OR clinically significant improvement OR clinical significant improvement OR clinically meaningful improvement OR clinical meaningful improvement OR clinically relevant improvement OR clinical relevant improvement OR clinically meaningful OR clinical meaningful OR meaningful improvement OR clinical significant difference OR clinically significant difference OR clinical important improvement OR clinically important improvement OR clinical meaningful change OR clinically meaningful change OR mcid OR minimally clinical important OR minimally clinically important OR minimum clinical important OR minimum clinically important OR minimal clinical important OR minimal clinically important OR minimum clinical detectable OR minimum clinically detectable OR minimal clinical detectable OR minimal clinically detectable OR minimum clinical significant OR minimum clinically significant OR minimal clinical significant OR minimal clinically significant OR minimum detectable difference OR minimum detectable difference OR minimal detectable difference OR minimal detectable difference OR minimum important change OR minimum important change OR minimal important change OR minimal important change OR minimum important difference OR minimum important difference OR minimal important difference OR minimal important difference OR smallest real difference OR subjectively significant difference)  11. 4 OR 5 OR 6 OR 7  12. 8 OR 9 OR 10  13. 3 AND 11 AND 12 |

^††^ “*”, “# “, and “?” are truncation characters that retrieve all possible suffix variations of the root word, e.g., urin* retrieves urinary, urine, etc.
