# Supplementary material for: The minimal important difference of patient-reported outcome measures related to female urinary incontinence: a systematic review
Source: BMC Med Res Methodol. 2024 Mar 8;24:60. doi: 10.1186/s12874-024-02188-4 (PMC10921720; doi:10.1186/s12874-024-02188-4)
Supplement: Supplementary file 3 — Supplementary Material 3. [file 12874_2024_2188_MOESM3_ESM.docx]

**Appendix 3.** Reasons for excluding studies during the full-text screening.

| Other study design (n=9)  Commentary   1. Wein 2017. Re: ICIQ Symptom and Quality of Life Instruments Measure Clinically Relevant Improvements in Women with Stress Urinary Incontinence   Conference papers   1. Karaman 2016. Is there a minimum important difference in outcomes of common validated questionnaires after sling surgery? 2. Brubaker 2013. The minimum clinically important difference for the international consultation on incontinence questionnaire (ICIQ) in women with stress urinary incontinence 3. Salvatore 2014. Long-term efficacy, safety, and quality of life analysis of repeated onabotulinumtoxina treatment for patients with idiopathic overactive bladder syndrome and urinary incontinence 4. Sand 2014. Long-term improvements in quality of life following onabotulinumtoxina treatment in female patients with overactive bladder and urinary incontinence 5. Sherburn 2009. Evaluation of outcome measures for stress urinary incontinence in older women 6. Sirls 2013. The minimum clinically important difference for the international consultation on incontinence questionnaire (ICIQ) in women with stress urinary incontinence 7. Tannenbaum 2013. Challenges in determining the cost-utility of incontinence treatment: An analysis of the problem and possible solutions   Review   1. Homma 2002. The clinical significance of the urodynamic investigation in incontinence |
| --- |
| Urinary incontinence was not the condition of interest (n=5)   1. Teig CJ, Grotle M, Bond MJ, Prinsen CAC, Engh MAE, Cvancarova MS, Kjøllesdal M, Martini A. Norwegian translation, and validation, of the Pelvic Floor Distress Inventory (PFDI-20) and the Pelvic Floor Impact Questionnaire (PFIQ-7). Int Urogynecol J. 2017 Jul;28(7):1005-1017. doi: 10.1007/s00192-016-3209-z. Epub 2017 Jan 6. 2. Mao M, Ai F, Zhang Y, Kang J, Liang S, Xu T, Zhu L. Changes in the symptoms and quality of life of women with symptomatic pelvic organ prolapse fitted with a ring with support pessary. Maturitas. 2018 Nov;117:51-56. doi: 10.1016/j.maturitas.2018.09.003. Epub 2018 Sep 19. 3. Clayson D, Wild D, Doll H, Keating K, Gondek K. Validation of a patient-administered questionnaire to measure the severity and bothersomeness of lower urinary tract symptoms in uncomplicated urinary tract infection (UTI): the UTI Symptom Assessment questionnaire. BJU Int. 2005 Aug;96(3):350-9. doi: 10.1111/j.1464-410X.2005.05630.x. 4. Wiegersma M, Panman CM, Berger MY, De Vet HC, Kollen BJ, Dekker JH. Minimal important change in the pelvic floor distress inventory-20 among women opting for conservative prolapse treatment. Am J Obstet Gynecol. 2017 Apr;216(4):397.e1-397.e7. doi: 10.1016/j.ajog.2016.10.010. Epub 2016 Oct 15. 5. Mamik MM, Rogers RG, Qualls CR, Morrow JD. The minimum important difference for the Pelvic Organ Prolapse-Urinary Incontinence Sexual Function Questionnaire. Int Urogynecol J. 2014 Oct;25(10):1321-6. doi: 10.1007/s00192-014-2342-9. Epub 2014 Feb 22. |
| Study did not reported measurements of minimal important difference (n=16)   1. Brucker BM, Jericevic D, Rude T, Enemchukwu E, Pape D, Rosenblum N, Charlson ER, Zhovtis-Ryerson L, Howard J, Krupp L, Peyronnet B. Mirabegron Versus Solifenacin in Multiple Sclerosis Patients With Overactive Bladder Symptoms: A Prospective Comparative Nonrandomized Study. Urology. 2020 Nov;145:94-99. doi: 10.1016/j.urology.2020.08.008. Epub 2020 Aug 19. 2. Yalcin, Ilker, G. Peng, Lars Viktrup and Richard C. Bump. “Reductions in stress urinary incontinence episodes: What is clinically important for Women?” Neurourology and Urodynamics 29 (2010): n. pag. 3. Uebersax JS, Wyman JF, Shumaker SA, McClish DK, Fantl JA. Short forms to assess life quality and symptom distress for urinary incontinence in women: the Incontinence Impact Questionnaire and the Urogenital Distress Inventory. Continence Program for Women Research Group. Neurourol Urodyn. 1995;14(2):131-9. doi: 10.1002/nau.1930140206. 4. Wijma J, Weis Potters AE, Tinga DJ, Aarnoudse JG. The diagnostic strength of the 24-h pad test for self-reported symptoms of urinary incontinence in pregnancy and after childbirth. Int Urogynecol J Pelvic Floor Dysfunct. 2008 Apr;19(4):525-30. doi: 10.1007/s00192-007-0472-z. Epub 2007 Oct 10 5. MacDiarmid S, Al-Shukri S, Barkin J, Fianu-Jonasson A, Grise P, Herschorn S, Saleem T, Huang M, Siddiqui E, Stölzel M, Hemsted C, Nazir J, Hakimi Z, Drake MJ; BESIDE Investigators. Mirabegron as Add-On Treatment to Solifenacin in Patients with Incontinent Overactive Bladder and an Inadequate Response to Solifenacin Monotherapy: Responder Analyses and Patient-Reported Outcomes from the BESIDE Study [corrected]. J Urol. 2016 Sep;196(3):809-18. doi: 10.1016/j.juro.2016.03.174. Epub 2016 Apr 8. Erratum in: J Urol. 2016 Dec;196(6):1826.. 6. Harvie HS, Shea JA, Andy UU, Propert K, Schwartz JS, Arya LA. Validity of utility measures for women with urge, stress, and mixed urinary incontinence. Am J Obstet Gynecol. 2014 Jan;210(1):85.e1-6. doi: 10.1016/j.ajog.2013.09.025. Epub 2013 Sep 18. 7. Lowenstein L, Kenton K, FitzGerald MP, Brubaker L. Clinically useful measures in women with mixed urinary incontinence. Am J Obstet Gynecol. 2008 Jun;198(6):664.e1-3; discussion 664.e3-4. doi: 10.1016/j.ajog.2008.02.014. 8. Kallner HK, Elmér C, Andersson KE, Altman D. Hormonal influence on the effect of mirabegron treatment for overactive bladder. Menopause. 2016 Dec;23(12):1303-1306. doi: 10.1097/GME.0000000000000708. 9. Colman S, Chapple C, Nitti V, Haag-Molkenteller C, Hastedt C, Massow U. Validation of treatment benefit scale for assessing subjective outcomes in treatment of overactive bladder. Urology. 2008 Oct;72(4):803-7. doi: 10.1016/j.urology.2008.05.033. Epub 2008 Aug 22. 10. Erel CT, Fistonić I, Gambacciani M, Oner Y, Fistonić N. Er:YAG laser in hysterectomized women with stress urinary incontinence: a VELA retrospective cohort, non-inferiority study. Climacteric. 2020;23(sup1):S18-S23. doi: 10.1080/13697137.2020.1814728. 11. Frick AC, Ridgeway B, Ellerkmann M, Karram MM, Paraiso MF, Walters MD, Barber MD. Comparison of responsiveness of validated outcome measures after surgery for stress urinary incontinence. J Urol. 2010 Nov;184(5):2013-7. doi: 10.1016/j.juro.2010.06.114. Epub 2010 Sep 17. 12. Arya LA, Heidi H, Cory L, Segal S, Northington GM. Construct validity of a questionnaire to measure the type of fluid intake and type of urinary incontinence. Neurourol Urodyn. 2011 Nov;30(8):1597-602. doi: 10.1002/nau.21091. Epub 2011 Apr 1. 13. Voigt R, Al-Hasan A, Voigt P, Halaska M. Zur Beziehung zwischen statischem Urethradruckprofil und Harndrangsymptomatik der Frau [Relation of the static urethral pressure profile and symptoms of urinary urgency in the female]. Z Urol Nephrol. 1988 Jun;81(6):345-9. German. 14. Suskind AM, Dunn RL, Morgan DM, DeLancey JO, Rew KT, Wei JT. A screening tool for clinically relevant urinary incontinence. Neurourol Urodyn. 2015 Apr;34(4):332-5. doi: 10.1002/nau.22564. Epub 2014 Jan 25. 15. Gottlieb D, Dvir Z, Golomb J, Beer-Gabel M. Reproducibility of ultrasonic measurements of pelvic floor structures in women suffering from urinary incontinence. Int Urogynecol J Pelvic Floor Dysfunct. 2009 Mar;20(3):309-12. doi: 10.1007/s00192-008-0771-z. Epub 2008 Nov 22. 16. Bates D, Burks J, Globe D, Signori M, Hudgens S, Denys P, Macdiarmid S, Nitti V, Odderson I, Ross AP, Chancellor M. Development of a short form and scoring algorithm from the validated actionable bladder symptom screening tool. BMC Neurol. 2013 Jul 9;13:78. doi: 10.1186/1471-2377-13-78. |
| Men and women included in the same sample (n=6)   1. Schurch B, Denys P, Kozma CM, Reese PR, Slaton T, Barron R. Reliability and validity of the Incontinence Quality of Life questionnaire in patients with neurogenic urinary incontinence. Arch Phys Med Rehabil. 2007 May;88(5):646-52. doi: 10.1016/j.apmr.2007.02.009. 2. Utomo E, Korfage IJ, Wildhagen MF, Steensma AB, Bangma CH, Blok BF. Validation of the Urogenital Distress Inventory (UDI-6) and Incontinence Impact Questionnaire (IIQ-7) in a Dutch population. Neurourol Urodyn. 2015 Jan;34(1):24-31. doi: 10.1002/nau.22496. Epub 2013 Oct 26. 3. Abrams P, Kelleher C, Huels J, Quebe-Fehling E, Omar MA, Steel M. Clinical relevance of health-related quality of life outcomes with darifenacin. BJU Int. 2008 Jul;102(2):208-13. doi: 10.1111/j.1464-410X.2008.07523.x. Epub 2008 Jul 1. 4. Gotoh M, Homma Y, Yokoyama O, Nishizawa O. Responsiveness and minimal clinically important change in overactive bladder symptom score. Urology. 2011 Oct;78(4):768-73. doi: 10.1016/j.urology.2011.06.020. 5. Kelleher CJ, Pleil AM, Reese PR, Burgess SM, Brodish PH. How much is enough and who says so? BJOG. 2004 Jun;111(6):605-12. doi: 10.1111/j.1471-0528.2004.00129.x. 6. Homma Y, Koyama N. Minimal clinically important change in urinary incontinence detected by a quality of life assessment tool in overactive bladder syndrome with urge incontinence. Neurourol Urodyn. 2006;25(3):228-235. doi: 10.1002/nau.20195. |
| Authors included women with other PFM dysfunctions did not reported the MID for participants only with UI separately (n=1)  Ma Y, Xu T, Zhang Y, Mao M, Kang J, Zhu L. Validation of the Chinese version of the Pelvic Floor Distress Inventory-20 (PFDI-20) according to the COSMIN checklist. Int Urogynecol J. 2019 Jul;30(7):1127-1139. doi: 10.1007/s00192-018-3847-4. Epub 2019 Feb 4. |
| Duplicate (n=3)   1. Kelleher CJ, Pleil AM, Reese PR, Burgess SM, Brodish PH. How much is enough and who says so? BJOG. 2004 Jun;111(6):605-12. doi: 10.1111/j.1471-0528.2004.00129.x. PMID: 15198790. 2. Morgan DM, Umek W, Guire K, Morgan HK, Garabrant A, DeLancey JO. Urethral sphincter morphology and function with and without stress incontinence. *J Urol*. 2009;182(1):203-209. doi:10.1016/j.juro.2009.02.129 3. Barber MD, Spino C, Janz NK, Brubaker L, Nygaard I, Nager CW, Wheeler TL; Pelvic Floor Disorders Network. The minimum important differences for the urinary scales of the Pelvic Floor Distress Inventory and Pelvic Floor Impact Questionnaire. Am J Obstet Gynecol. 2009 May;200(5):580.e1-7. doi: 10.1016/j.ajog.2009.02.007. |
| Study was conducted with continent women (n=2)   1. Castro-Diaz D, Chapple CR, Hakimi Z, Blauwet MB, Delgado-Herrera L, Lau W, Mujais S. The effect of mirabegron on patient-related outcomes in patients with overactive bladder: the results of post hoc correlation and responder analyses using pooled data from three randomized Phase III trials. Qual Life Res. 2015 Jul;24(7):1719-27. doi: 10.1007/s11136-014-0904-4. Epub 2015 Feb 17. 2. Coyne KS, Matza LS, Thompson CL, Kopp ZS, Khullar V. Determining the importance of change in the overactive bladder questionnaire. J Urol. 2006 Aug;176(2):627-32; discussion 632. doi: 10.1016/j.juro.2006.03.088. |
