# Supplementary material for: The minimal important difference of patient-reported outcome measures related to female urinary incontinence: a systematic review
Source: BMC Med Res Methodol. 2024 Mar 8;24:60. doi: 10.1186/s12874-024-02188-4 (PMC10921720; doi:10.1186/s12874-024-02188-4)
Supplement: Supplementary file 4 — Supplementary Material 4. [file 12874_2024_2188_MOESM4_ESM.docx]

**Appendix 4**. Assessment of the credibility according to the MID tool, proposed by Devil et al. 2020.

| ***Nº*** | ***Articles*** | ***PROM*** | ***Anchor*** | ***Follow-up*** | **Item 1** | **Item 2** | **Item 3** | **Item 4** | **Item 5** | **Item 6** | Guide | **Item 7** | **Item 8** | **Item 9** | Final score |
| --- | --- | --- | --- | --- | --- | --- | --- | --- | --- | --- | --- | --- | --- | --- | --- |
| 1.1 | Baessler et al. | Australian Pelvic Floor Questionnaire - Bladder | Patient Global Impression of Improvement questionnaire | NA | Yes | To a great extent | General correlation provided with not exact correlation | Definitely no (≥50% or <100 patients) | Definitely yes | To a great extent (>1 to ≤2 months) | Guide B | Impossible to tell | Definitely yes | Impossible to tell | Low |
| 1.2 | Baessler et al. | Australian Pelvic Floor Questionnaire - Global | Patient Global Impression of Improvement questionnaire | NA | Yes | To a great extent | General correlation provided with not exact correlation | Definitely no (≥50% or <100 patients) | Definitely yes | To a great extent (>1 to ≤2 months) | Guide B | Impossible to tell | Definitely yes | Impossible to tell | Low |
| 1.3 | Yalcin et al., | Incontinence Quality of Life (I-QOL) - Within-treatment (mean, SD) - Total score | Patient Global Impression of Improvement questionnaire | NA | Yes | Definitely yes | Impossible to tell | Not so much (26-49% or 100-149 patients) | Definitely yes | Not so much (>2 to ≤3 months) | Guide A | Impossible to tell | Impossible to tell | Impossible to tell | Low |
| 1.4 | Patrick et al | Incontinence Quality of Life (I-QOL) | Patient Global Perception of Change | NA | Yes | Definitely yes | Impossible to tell | Definitely no (≥50% or <100 patients) | Definitely yes | Not so much (>2 to ≤3 months) | Guide B | Impossible to tell | Impossible to tell | Impossible to tell | Low |
| 1.5 | Yalcin et al., | Incontinence Quality of Life (I-QOL) - Within-treatment (mean, SD) - Avoidance and Limiting Behavior | Patient Global Impression of Improvement questionnaire | NA | Yes | Definitely yes | Impossible to tell | Not so much (26-49% or 100-149 patients) | Definitely yes | Not so much (>2 to ≤3 months) | Guide A | Impossible to tell | Impossible to tell | Impossible to tell | Low |
| 1.6 | Yalcin et al., | Incontinence Quality of Life (I-QOL) - Within-treatment (mean, SD) - Psychosocial Impacts | Patient Global Impression of Improvement questionnaire | NA | Yes | Definitely yes | Impossible to tell | Not so much (26-49% or 100-149 patients) | Definitely yes | Not so much (>2 to ≤3 months) | Guide A | Impossible to tell | Impossible to tell | Impossible to tell | Low |
| 1.7 | Yalcin et al., | Incontinence Quality of Life (I-QOL) - Within-treatment (mean, SD) - Social Embarassement | Patient Global Impression of Improvement questionnaire | NA | Yes | Definitely yes | Impossible to tell | Definitely no (≥50% or <100 patients) | Definitely yes | Not so much (>2 to ≤3 months) | Guide A | Impossible to tell | Impossible to tell | Impossible to tell | Low |
| 1.8 | Patrick et al | Incontinence Quality of Life (I-QOL) | Pad Test | NA | No | Not so much | Impossible to tell | Not so much (26-49% or 100-149 patients) | Definitely no | Not so much (>2 to ≤3 months) | Guide B | Impossible to tell | Impossible to tell | Impossible to tell | Low |
| 1.9 | Patrick et al | Incontinence Quality of Life (I-QOL) | Voiding diary | NA | Yes | To a great extent | Impossible to tell | Definitely no (≥50% or <100 patients) | Definitely no | Not so much (>2 to ≤3 months) | Guide B | Impossible to tell | Impossible to tell | Impossible to tell | Low |
| 1.10 | Lim et al | International Consultation on Incontinence Questionnaire - Short Form (ICIQ-SF) | Patient Global Impression of Improvement questionnaire | NA | Yes | To a great extent | General correlation provided with not exact correlation | Definitely no (≥50% or <100 patients) | Definitely no | Definitely no (>3 months) | Guide B | Impossible to tell | Definitely yes | Impossible to tell | Low |
| 1.11 | Nystrom et al. | International Consultation on Incontinence Questionnaire - Short Form (ICIQ-SF) | Patient Global Impression of Improvement questionnaire | NA | Yes | Definitely yes | Impossible to tell | Not so much (26-49% or 100-149 patients) | Definitely yes | Definitely no (>3 months) | Guide B | Impossible to tell | Impossible to tell | Impossible to tell | Low |
| 1.12 | Sirls et al | International Consultation on Incontinence Questionnaire - Short Form (ICIQ-SF) | Patient Global Impression of Improvement questionnaire | 12 months | Yes | To a great extent | General correlation provided with not exact correlation | Not so much (26-49% or 100-149 patients) | Definitely no | Definitely no (>3 months) | Guide B | Definitely no | Definitely yes | Not so much | Low |
| 1.13 | Sirls et al | International Consultation on Incontinence Questionnaire - Short Form (ICIQ-SF) | Patient Global Impression of Improvement questionnaire | 24 months | Yes | To a great extent | General correlation provided with not exact correlation | Not so much (26-49% or 100-149 patients) | Definitely no | Definitely no (>3 months) | Guide B | Definitely no | Definitely yes | Not so much | Low |
| 1.14 | Nipa et al., | International Consultation on Incontinence Questionnaire - Short Form (ICIQ-SF) | Patient Global Impression of Improvement questionnaire | 3 months: SIMS trial | Yes | To a great extent | Impossible to tell | Definitely yes (≤10% or ≥200 patients) | To a great extent | Not so much (>2 to ≤3 months) | Guide B | Impossible to tell | Impossible to tell | Impossible to tell |  |
| 1.15 | Nipa et al., | International Consultation on Incontinence Questionnaire - Short Form (ICIQ-SF) | Patient Global Impression of Improvement questionnaire | 1 year: SIMS trial | Yes | To a great extent | Impossible to tell | Definitely yes (≤10% or ≥200 patients) | To a great extent | Definitely no (>3 months) | Guide B | Impossible to tell | Impossible to tell | Impossible to tell | Yes |
| 1.16 | Nipa et al., | International Consultation on Incontinence Questionnaire - Short Form (ICIQ-SF) | Patient Global Impression of Improvement questionnaire | 2 years: SIMS trial | Yes | To a great extent | Impossible to tell | Definitely yes (≤10% or ≥200 patients) | To a great extent | Definitely no (>3 months) | Guide B | Impossible to tell | Impossible to tell | Impossible to tell | Yes |
| 1.17 | Nipa et al., | International Consultation on Incontinence Questionnaire - Short Form (ICIQ-SF) | Patient Global Impression of Improvement questionnaire | 3 years: SIMS trial | Yes | To a great extent | Impossible to tell | Definitely yes (≤10% or ≥200 patients) | To a great extent | Definitely no (>3 months) | Guide B | Impossible to tell | Impossible to tell | Impossible to tell | Yes |
| 1.18 | Nipa et al., | International Consultation on Incontinence Questionnaire - Short Form (ICIQ-SF) | Patient Global Impression of Improvement questionnaire | 6 months: OPAL trial | Yes | To a great extent | Impossible to tell | Definitely yes (≤10% or ≥200 patients) | To a great extent | Definitely no (>3 months) | Guide B | Impossible to tell | Impossible to tell | Impossible to tell | Yes |
| 1.19 | Nipa et al., | International Consultation on Incontinence Questionnaire - Short Form (ICIQ-SF) | Patient Global Impression of Improvement questionnaire | 1-year: OPAL trial | Yes | To a great extent | Impossible to tell | Definitely yes (≤10% or ≥200 patients) | To a great extent | Definitely no (>3 months) | Guide B | Impossible to tell | Impossible to tell | Impossible to tell | Yes |
| 1.20 | Nipa et al., | International Consultation on Incontinence Questionnaire - Short Form (ICIQ-SF) | Patient Global Impression of Improvement questionnaire | 2-years: OPAL trial | Yes | To a great extent | Impossible to tell | Definitely yes (≤10% or ≥200 patients) | To a great extent | Definitely no (>3 months) | Guide B | Impossible to tell | Impossible to tell | Impossible to tell | Yes |
| 1.21 | Lim et al | International Consultation on Incontinence Questionnaire - Short Form (ICIQ-SF) | Satisfaction with the treatment | NA | Yes | To a great extent | General correlation provided with not exact correlation | Not so much (26-49% or 100-149 patients) | Not so much | Definitely no (>3 months) | Guide B | Impossible to tell | Definitely yes | Impossible to tell | Low |
| 1.22 | Sirls et al | International Consultation on Incontinence Questionnaire - Short Form (ICIQ-SF) | Satisfaction with the treatment | 12 months | Yes | Definitely yes | General correlation provided with not exact correlation | Definitely no (≥50% or <100 patients) | Definitely no | Definitely no (>3 months) | Guide B | Definitely no | Definitely yes | Not so much | Low |
| 1.23 | Sirls et al | International Consultation on Incontinence Questionnaire - Short Form (ICIQ-SF) | Satisfaction with the treatment | 24 months | Yes | Definitely yes | General correlation provided with not exact correlation | Definitely no (≥50% or <100 patients) | Definitely no | Definitely no (>3 months) | Guide B | Definitely no | Definitely yes | Not so much | Low |
| 1.24 | Lim et al | International Consultation on Incontinence Questionnaire - Short Form (ICIQ-SF) | Voiding diary | NA | Yes | To a great extent | General correlation provided with not exact correlation | Definitely no (≥50% or <100 patients) | Definitely no | Definitely no (>3 months) | Guide A | Impossible to tell | Definitely no | Impossible to tell | Low |
| 1.25 | Sirls et al | International Consultation on Incontinence Questionnaire - Short Form (ICIQ-SF) | Voiding diary - 25% | 12 months | Yes | To a great extent | General correlation provided with not exact correlation | Definitely no (≥50% or <100 patients) | Definitely no | Definitely no (>3 months) | Guide A | Definitely yes | Definitely no | Not so much | Low |
| 1.26 | Sirls et al | International Consultation on Incontinence Questionnaire - Short Form (ICIQ-SF) | Voiding diary - 25% | 24 months | Yes | To a great extent | General correlation provided with not exact correlation | Definitely no (≥50% or <100 patients) | Definitely no | Definitely no (>3 months) | Guide A | Definitely yes | Definitely no | Not so much | Low |
| 1.27 | Lim et al | International Consultation on Incontinence Questionnaire - Short Form (ICIQ-SF) | 1-h pad test | NA | No (the patient does not know how much she is leaking) | Not so much | General correlation provided with not exact correlation | Definitely no (≥50% or <100 patients) | Definitely no | Definitely no (>3 months) | Guide A | Impossible to tell | Definitely no | Impossible to tell | Low |
| 1.28 | Sirls et al | International Consultation on Incontinence Questionnaire - Short Form (ICIQ-SF) | Urogenital Distress Inventory (UDI) | 12 months | Yes | To a great extent | General correlation provided with not exact correlation | Definitely no (≥50% or <100 patients) | Definitely no | Definitely no (>3 months) | Guide A | Definitely yes | Definitely no | Definitely no | Low |
| 1.29 | Sirls et al | International Consultation on Incontinence Questionnaire - Short Form (ICIQ-SF) | Urogenital Distress Inventory (UDI) | 24 months | Yes | To a great extent | General correlation provided with not exact correlation | Not so much (26-49% or 100-149 patients) | Definitely no | Definitely no (>3 months) | Guide A | Definitely yes | Definitely no | Definitely no | Low |
| 1.30 | Sirls et al | International Consultation on Incontinence Questionnaire - Short Form (ICIQ-SF) | Incontinence Impact Questionnaire (IIQ) | 12 months | Yes | To a great extent | General correlation provided with not exact correlation | Not so much (26-49% or 100-149 patients) | Definitely no | Definitely no (>3 months) | Guide A | Definitely yes | Definitely no | Not so much | Low |
| 1.31 | Sirls et al | International Consultation on Incontinence Questionnaire - Short Form (ICIQ-SF) | Incontinence Impact Questionnaire (IIQ) | 24 months | Yes | To a great extent | General correlation provided with not exact correlation | Definitely no (≥50% or <100 patients) | Definitely no | Definitely no (>3 months) | Guide A | Definitely yes | Definitely no | Not so much | Low |
| 1.32 | Lim et al | ICIQ-Lower Urinary Tract Symptoms Quality of Life (ICIQ-LUTSqol) | Patient Global Impression of Improvement questionnaire | NA | Yes | To a great extent | General correlation provided with not exact correlation | Definitely no (≥50% or <100 patients) | Definitely no | Definitely no (>3 months) | Guide B | Impossible to tell | Definitely yes | Impossible to tell | Low |
| 1.33 | Nystrom et al. | ICIQ-Lower Urinary Tract Symptoms Quality of Life (ICIQ-LUTSqol) | Patient Global Impression of Improvement questionnaire | NA | Yes | Definitely yes | Impossible to tell | Not so much (26-49% or 100-149 patients) | Definitely yes | Definitely no (>3 months) | Guide B | Impossible to tell | Impossible to tell | Impossible to tell | Low |
| 1.34 | Lim et al | ICIQ-Lower Urinary Tract Symptoms Quality of Life (ICIQ-LUTSqol) | Satisfaction with the treatment | NA | Yes | To a great extent | General correlation provided with not exact correlation | Definitely no (≥50% or <100 patients) | Not so much | Definitely no (>3 months) | Guide B | Impossible to tell | Definitely yes | Impossible to tell | Low |
| 1.35 | Lim et al | ICIQ-Lower Urinary Tract Symptoms Quality of Life (ICIQ-LUTSqol) | Voiding diary | NA | Yes | To a great extent | General correlation provided with not exact correlation | Definitely no (≥50% or <100 patients) | Definitely no | Definitely no (>3 months) | Guide A | Impossible to tell | Definitely no | Impossible to tell | Low |
| 1.36 | Lim et al | ICIQ-Lower Urinary Tract Symptoms Quality of Life (ICIQ-LUTSqol) | 1-h pad test | NA | No | Not so much | General correlation provided with not exact correlation | Definitely no (≥50% or <100 patients) | Definitely no | Definitely no (>3 months) | Guide A | Impossible to tell | Definitely no | Impossible to tell | Low |
| 1.37 | Barber et al., | UIQ | Patient Global Impression of Improvement questionnaire | NA | Yes | Definitely yes | General correlation provided with not exact correlation | Definitely no (≥50% or <100 patients) | Definitely yes | Not so much (>2 to ≤3 months) | Guide B | Impossible to tell | Impossible to tell | Impossible to tell | Low |
| 1.38 | Chan et al., | UIQ subscale of PFIQ | Self-reported satisfaction with the treatment received (very satisfied, moderately satisfied, somewhat satisfied, or dissatisfied) | NA | Yes | To a great extend | Impossible to tell | Definitely no (≥50% or <100 patients) | Not so much | Not so much (>2 to ≤3 months) | Guide B | Impossible to tell | Impossible to tell | Impossible to tell | Low |
| 1.39 | Barber et al., | UIQ | Voiding diary | NA | Yes | To a great extent | General correlation provided with not exact correlation | Definitely no (≥50% or <100 patients) | Definitely no | Not so much (>2 to ≤3 months) | Guide B | Impossible to tell | Definitely no | Impossible to tell | Low |
| 1.40 | Barber et al., | UIQ | Incontinence Severity Index | NA | Yes | To a great extent | General correlation provided with not exact correlation | Definitely no (≥50% or <100 patients) | Definitely yes | Not so much (>2 to ≤3 months) | Guide A | Impossible to tell | Definitely no | Impossible to tell | Low |
| 1.41 | Chan et al., | UIQ subscale of PFIQ | 10-cm VAS score indicating the severity of symptoms | NA | Yes | To a great extend | To a great extent (≥0.5 to <0.7) | Definitely no (≥50% or <100 patients) | To a great extend | Not so much (>2 to ≤3 months) |  | Definitely yes | Definitely no | Definitely yes | Low |
| 1.42 | Barber et al., | Urogenital Distress Inventory (UDI) | Patient Global Impression of Improvement questionnaire | NA | Yes | Definitely yes | General correlation provided with not exact correlation | Definitely no (≥50% or <100 patients) | Definitely yes | Not so much (>2 to ≤3 months) | Guide B | Impossible to tell | Impossible to tell | Impossible to tell | Low |
| 1.43 | Dyer et al | Urogenital Distress Inventory (UDI) | Global Perception of Improvement | 10 weeks | Yes | Definitely yes | General correlation provided with not exact correlation | Definitely no (≥50% or <100 patients) | To a great extent | Not so much | Guide B | Definitely no | Definitely yes | To a great extent | Low |
| 1.44 | Dyer et al | Urogenital Distress Inventory (UDI) | Global Perception of Improvement | 8 months | Yes | Definitely yes | General correlation provided with not exact correlation | Definitely no (≥50% or <100 patients) | To a great extent | Definitely no (>3 months) | Guide B | Definitely no | Definitely yes | To a great extent | Low |
| 1.45 | Dyer et al | Urogenital Distress Inventory (UDI) | Patient Satisfaction Questionnaire | 10 weeks | Yes | Definitely yes | General correlation provided with not exact correlation | Definitely no (≥50% or <100 patients) | Not so much | Not so much | Guide B | Definitely no | Definitely yes | To a great extent | Low |
| 1.46 | Dyer et al | Urogenital Distress Inventory (UDI) | Patient Satisfaction Questionnaire | 8 months | Yes | Definitely yes | General correlation provided with not exact correlation | Definitely no (≥50% or <100 patients) | Not so much | Definitely no (>3 months) | Guide B | Definitely no | Definitely yes | To a great extent | Low |
| 1.47 | Barber et al., | Urogenital Distress Inventory (UDI) | Incontinence Severity Index | NA | Yes | To a great extent | General correlation provided with not exact correlation | Definitely no (≥50% or <100 patients) | Definitely yes | Not so much (>2 to ≤3 months) | Guide A | Impossible to tell | Definitely no | Impossible to tell | Low |
| 1.48 | Chan et al., | UDI subscale of PFDI | Self-reported satisfaction with the treatment received (very satisfied, moderately satisfied, somewhat satisfied or dissatisfied) | NA | Yes | To a great extend | Impossible to tell | Definitely no (≥50% or <100 patients) | Not so much | Not so much (>2 to ≤3 months) | Guide B | Impossible to tell | Impossible to tell | Impossible to tell | Low |
| 1.49 | Barber et al., | Urogenital Distress Inventory (UDI) | Voiding diary | NA | Yes | To a great extent | General correlation provided with not exact correlation | Definitely no (≥50% or <100 patients) | Definitely no | Not so much (>2 to ≤3 months) | Guide B | Impossible to tell | Definitely no | Impossible to tell | Low |
| 1.50 | Dyer et al | Urogenital Distress Inventory (UDI) | Voiding diary - 25% | 10 weeks | Yes | To a great extend | General correlation provided with not exact correlation | Definitely no (≥50% or <100 patients) | Definitely no | Not so much | Guide B | Definitely no | Definitely yes | Not so much | Low |
| 1.51 | Dyer et al | Urogenital Distress Inventory (UDI) | Voiding diary - 25% | 8 months | Yes | To a great extend | General correlation provided with not exact correlation | Definitely no (≥50% or <100 patients) | Definitely no | Definitely no (>3 months) | Guide B | Definitely no | Definitely yes | Not so much | Low |
| 1.52 | Dyer et al | Urogenital Distress Inventory (UDI) | Voiding diary - 75% | 8 months | Yes | To a great extend | General correlation provided with not exact correlation | Definitely no (≥50% or <100 patients) | Definitely no | Definitely no (>3 months) | Guide B | Definitely no | Definitely yes | Not so much | Low |
| 1.53 | Chan et al., | UDI subscale of PFDI | 10-cm VAS score indicating the severity of symptoms | NA | Yes | To a great extend | To a great extent (≥0.5 to <0.7) | Definitely no (≥50% or <100 patients) | To a great extend | Not so much (>2 to ≤3 months) |  | Definitely yes | Definitely no | Not so much | Low |
| 1.54 | Dyer et al | UDI - Irritative Subscale | Global Perception of Improvement | 10 weeks | Yes | Definitely yes | General correlation provided with not exact correlation | Definitely no (≥50% or <100 patients) | To a great extent | Not so much | Guide B | Definitely no | Definitely yes | To a great extent | Low |
| 1.55 | Dyer et al | UDI - Irritative Subscale | Global Perception of Improvement | 8 months | Yes | Definitely yes | General correlation provided with not exact correlation | Definitely no (≥50% or <100 patients) | To a great extent | Definitely no (>3 months) | Guide B | Definitely no | Definitely yes | To a great extent | Low |
| 1.56 | Dyer et al | UDI - Irritative Subscale | Patient Satisfaction Questionnaire | 10 weeks | Yes | Definitely yes | General correlation provided with not exact correlation | Definitely no (≥50% or <100 patients) | Not so much | Not so much | Guide B | Definitely no | Definitely yes | To a great extent | Low |
| 1.57 | Dyer et al | UDI - Irritative Subscale | Patient Satisfaction Questionnaire | 8 months | Yes | Definitely yes | General correlation provided with not exact correlation | Definitely no (≥50% or <100 patients) | Not so much | Definitely no (>3 months) | Guide B | Definitely no | Definitely yes | To a great extent | Low |
| 1.58 | Dyer et al | UDI - Irritative Subscale | Voiding diary - 25% | 10 weeks | Yes | To a great extend | General correlation provided with not exact correlation | Definitely no (≥50% or <100 patients) | Definitely no | Not so much | Guide B | Definitely no | Definitely yes | Not so much | Low |
| 1.59 | Dyer et al | UDI - Irritative Subscale | Voiding diary - 25% | 8 months | Yes | To a great extend | General correlation provided with not exact correlation | Definitely no (≥50% or <100 patients) | Definitely no | Definitely no (>3 months) | Guide B | Definitely no | Definitely yes | Not so much | Low |
| 1.60 | Dyer et al | UDI - Irritative Subscale | Voiding diary - 75% | 10 weeks | Yes | To a great extend | General correlation provided with not exact correlation | Definitely no (≥50% or <100 patients) | Definitely no | Not so much | Guide B | Definitely no | Definitely yes | Not so much | Low |
| 1.61 | Dyer et al | UDI - Irritative Subscale | Voiding diary - 75% | 8 months | Yes | To a great extend | General correlation provided with not exact correlation | Definitely no (≥50% or <100 patients) | Definitely no | Definitely no (>3 months) | Guide B | Definitely no | Definitely yes | Not so much | Low |
| 1.62 | Barber et al., | Urogenital Distress Inventory (UDI) - Stress | Patient Global Impression of Improvement questionnaire | NA | Yes | Definitely yes | General correlation provided with not exact correlation | Definitely no (≥50% or <100 patients) | Definitely yes | Not so much (>2 to ≤3 months) | Guide B | Impossible to tell | Impossible to tell | Impossible to tell | Low |
| 1.63 | Barber et al., | Urogenital Distress Inventory (UDI) - Stress | Voiding diary | NA | Yes | To a great extent | General correlation provided with not exact correlation | Definitely no (≥50% or <100 patients) | Definitely no | Not so much (>2 to ≤3 months) | Guide B | Impossible to tell | Definitely no | Impossible to tell | Low |
| 1.64 | Barber et al., | Urogenital Distress Inventory (UDI) - Stress | Incontinence Severity Index | NA | Yes | To a great extent | General correlation provided with not exact correlation | Definitely no (≥50% or <100 patients) | Definitely yes | Not so much (>2 to ≤3 months) | Guide A | Impossible to tell | Definitely no | Impossible to tell | Low |
| 1.65 | Dyer et al | Overactive Bladder Questionnaire (OAB-q) | Global Perception of Improvement | 10 weeks | Yes | Definitely yes | General correlation provided with not exact correlation | Definitely no (≥50% or <100 patients) | To a great extent | Not so much | Guide A | Definitely yes | Definitely no | Definitely no | Low |
| 1.66 | Dyer et al | Overactive Bladder Questionnaire (OAB-q) | Global Perception of Improvement | 8 months | Yes | Definitely yes | General correlation provided with not exact correlation | Definitely no (≥50% or <100 patients) | To a great extent | Definitely no (>3 months) | Guide A | Definitely yes | Definitely no | Definitely no | Low |
| 1.67 | Dyer et al | Overactive Bladder Questionnaire (OAB-q) | Patient Satisfaction Questionnaire | 10 weeks | Yes | Definitely yes | General correlation provided with not exact correlation | Definitely no (≥50% or <100 patients) | Not so much | Not so much | Guide A | Definitely yes | Definitely no | Definitely no | Low |
| 1.68 | Dyer et al | Overactive Bladder Questionnaire (OAB-q) | Patient Satisfaction Questionnaire | 8 months | Yes | Definitely yes | General correlation provided with not exact correlation | Definitely no (≥50% or <100 patients) | Not so much | Definitely no (>3 months) | Guide A | Definitely yes | Definitely no | Definitely no | Low |
| 1.69 | Dyer et al | Overactive Bladder Questionnaire (OAB-q) | Voiding diary - 25% | 10 weeks | Yes | To a great extend | General correlation provided with not exact correlation | Definitely no (≥50% or <100 patients) | Definitely no | Not so much | Guide A | Definitely yes | Definitely no | Definitely no | Low |
| 1.70 | Dyer et al | Overactive Bladder Questionnaire (OAB-q) | Voiding diary - 25% | 8 months | Yes | To a great extend | General correlation provided with not exact correlation | Definitely no (≥50% or <100 patients) | Definitely no | Definitely no (>3 months) | Guide A | Definitely yes | Definitely no | Definitely no | Low |
| 1.71 | Dyer et al | Overactive Bladder Questionnaire (OAB-q) | Voiding diary - 75% | 10 weeks | Yes | To a great extend | General correlation provided with not exact correlation | Definitely no (≥50% or <100 patients) | Definitely no | Not so much | Guide A | Definitely yes | Definitely no | Not so much | Low |
| 1.72 | Dyer et al | Overactive Bladder Questionnaire (OAB-q) | Voiding diary - 75% | 8 months | Yes | To a great extend | General correlation provided with not exact correlation | Definitely no (≥50% or <100 patients) | Definitely no | Definitely no (>3 months) | Guide A | Definitely yes | Definitely no | Not so much | Low |
| 1.73 | Nipa et al., | International Consultation on  Incontinence Questionnaire – Female Lower Urinary Tract  Symptoms (ICIQ-FLUTS) | Patient Global Impression of Improvement questionnaire | 1 year: SIMS trial | Yes | To a great extent | Impossible to tell | Definitely yes (≤10% or ≥200 patients) | To a great extent | Definitely no (>3 months) | Guide B | Impossible to tell | Impossible to tell | Impossible to tell | 1.73- |
| 1.74 | Nipa et al., | International Consultation on  Incontinence Questionnaire – Female Lower Urinary Tract  Symptoms (ICIQ-FLUTS) | Patient Global Impression of Improvement questionnaire | 2 years: SIMS trial | Yes | To a great extent | Impossible to tell | Definitely yes (≤10% or ≥200 patients) | To a great extent | Definitely no (>3 months) | Guide B | Impossible to tell | Impossible to tell | Impossible to tell | Low |
| 1.75 | Nipa et al., | International Consultation on  Incontinence Questionnaire – Female Lower Urinary Tract  Symptoms (ICIQ-FLUTS) | Patient Global Impression of Improvement questionnaire | 3 years: SIMS trial | Yes | To a great extent | Impossible to tell | Definitely yes (≤10% or ≥200 patients) | To a great extent | Definitely no (>3 months) | Guide B | Impossible to tell | Impossible to tell | Impossible to tell | Low |
| 1.76 | Nipa et al., | International Consultation on  Incontinence Questionnaire – Female Lower Urinary Tract  Symptoms (ICIQ-FLUTS) | Patient Global Impression of Improvement questionnaire | 6 months: OPAL trial | Yes | To a great extent | Impossible to tell | Definitely yes (≤10% or ≥200 patients) | To a great extent | Definitely no (>3 months) | Guide B | Impossible to tell | Impossible to tell | Impossible to tell | Low |
| 1.77 | Nipa et al., | International Consultation on  Incontinence Questionnaire – Female Lower Urinary Tract  Symptoms (ICIQ-FLUTS) | Patient Global Impression of Improvement questionnaire | 1 year: OPAL trial | Yes | To a great extent | Impossible to tell | Definitely yes (≤10% or ≥200 patients) | To a great extent | Definitely no (>3 months) | Guide B | Impossible to tell | Impossible to tell | Impossible to tell | Low |
| 1.78 | Nipa et al., | International Consultation on  Incontinence Questionnaire – Female Lower Urinary Tract  Symptoms (ICIQ-FLUTS) | Patient Global Impression of Improvement questionnaire | 2 years: OPAL trial | Yes | To a great extent | Impossible to tell | Definitely yes (≤10% or ≥200 patients) | To a great extent | Definitely no (>3 months) | Guide B | Impossible to tell | Impossible to tell | Impossible to tell | Low |
|  | Total (Nº) | NA | NA | NA | Definitely yes = 78 | Definitely yes = 26  To a great extent = 49  Not so much = 3 | Impossible to tell= 24  General correlation= 52  To a great extent=2 | Definitely yes = 13  To a great extent = 1  Not so much = 6  Definitely no = 51  Impossible to tell = 4 | Definitely yes = 17  To a great extent = 19  Not so much = 16  Definitely no = 26 | Definitely yes =2  To a great extent = 13  Not so much = 21  Definitely no =42 | NA | Definitely yes = 8  Definitely no = 10  Impossible to tell = 60 | Definitely yes = 25  Definitely no = 26  Impossible to tell = 27 | Definitely yes = 5  To a great extend = 2  Not so much = 1  Definitely no = 10  Impossible to tell = 60 |  |

Description of items: Item 1) Is the patient or necessary proxy responding directly to both the PROM and the anchor?; Item 2) Is the anchor easily understandable and relevant for patients or necessary proxy?; Item 3) Has the anchor shown good correlation with the PROM?; Item 4) I s the MID precise?; Item 5) Does the threshold or difference between groups on the anchor used to estimate the MID reflect a small but important difference?; Item 6) Is the amount of elapsed time between baseline and follow-up measurement for MID estimation optimal?; Item 7) Does the transition item have a satisfactory correlation with the PROM score at follow-up?; Item 8) Does the transition item correlate with the PROM score at baseline?; Item 9) Is the correlation of the transition item with the PROM change score appreciably greater than the correlation of the transition item with the PROM score at follow-up?
