# Supplementary material for: The minimal important difference of patient-reported outcome measures related to female urinary incontinence: a systematic review
Source: BMC Med Res Methodol. 2024 Mar 8;24:60. doi: 10.1186/s12874-024-02188-4 (PMC10921720; doi:10.1186/s12874-024-02188-4)
Supplement: Supplementary file 5 — Supplementary Material 5. [file 12874_2024_2188_MOESM5_ESM.docx]

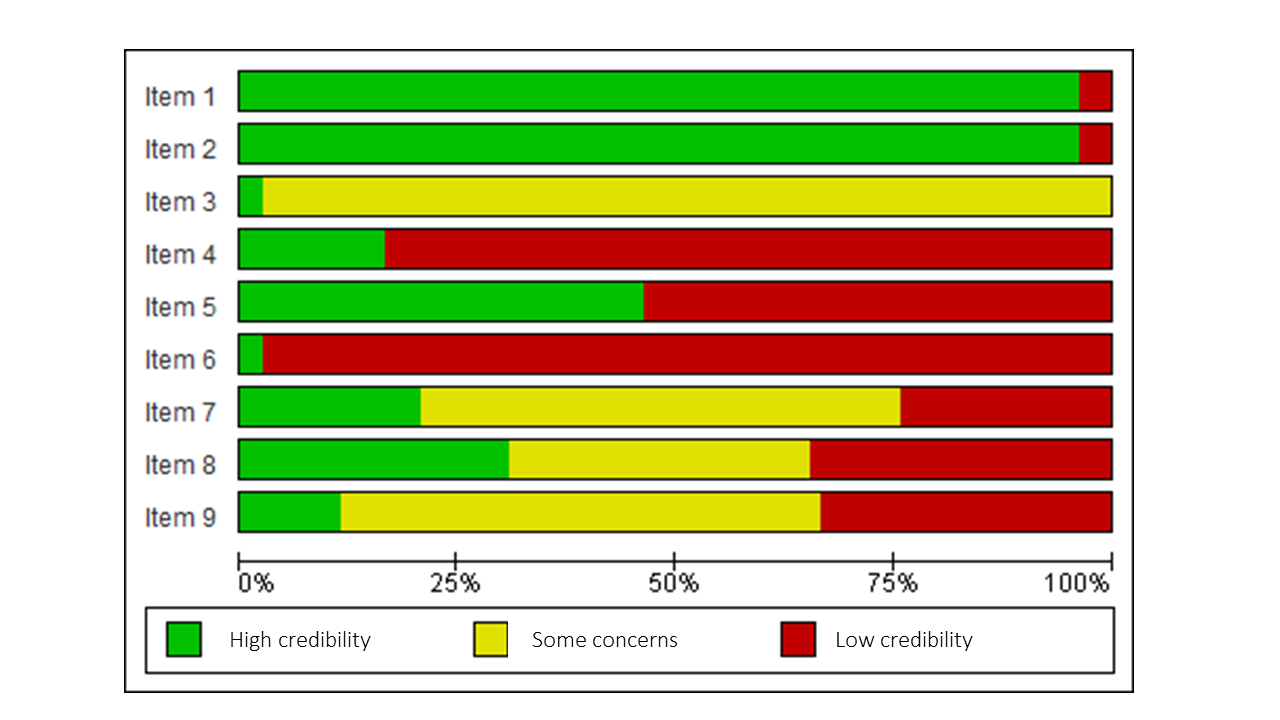


**Appendix 5.** Credibility graph: review authors' judgements about each credibility item presented as percentages across all included studies.

Description of items: Item 1) Is the patient or necessary proxy responding directly to both the PROM and the anchor?; Item 2) Is the anchor easily understandable and relevant for patients or necessary proxy?; Item 3) Has the anchor shown good correlation with the PROM?; Item 4) I s the MID precise?; Item 5) Does the threshold or difference between groups on the anchor used to estimate the MID reflect a small but important difference?; Item 6) Is the amount of elapsed time between baseline and follow-up measurement for MID estimation optimal?; Item 7) Does the transition item have a satisfactory correlation with the PROM score at follow-up?; Item 8) Does the transition item correlate with the PROM score at baseline?; Item 9) Is the correlation of the transition item with the PROM change score appreciably greater than the correlation of the transition item with the PROM score at follow-up?
