# Supplementary material for: The minimal important difference of patient-reported outcome measures related to female urinary incontinence: a systematic review
Source: BMC Med Res Methodol. 2024 Mar 8;24:60. doi: 10.1186/s12874-024-02188-4 (PMC10921720; doi:10.1186/s12874-024-02188-4)
Supplement: Supplementary file 7 — Supplementary Material 7. [file 12874_2024_2188_MOESM7_ESM.docx]

**Appendix 7.** Anchor-based methods calculations according to specific PROMs and anchors reported by the studies included in the present systematic review.

| **Author** | **PROM** | **Anchor** | **Correlation between PROM and anchor** | **Categories of the anchor** | **Description** | **Level of improvement used during data analysis** |
| --- | --- | --- | --- | --- | --- | --- |
| Barber et al.,(41) | Urogenital Distress Inventory (UDI) | Patient Global Impression of Improvement questionnaire | ≥ 0.3 | Participants could choose between these options: very much better, much better, better, about the same, worse, much worse, very much worse | Mean change in UDI score, UDI-stress and UIQ scales reported by women who indicated they were “better” at 3 months relative to the start of treatment and those who indicated they were “about the same” | Slight |
|  | Urogenital Distress Inventory (UDI) – Stress |  |  |  |  |  |
|  | UIQ |  |  |  |  |  |
|  | Urogenital Distress Inventory (UDI) | Incontinence Severity Index | ≥ 0.3 | The final score was calculated by multiplying the incontinence frequency by the amount of leakage. Based on the resultant ISI value (0–12) subjects were further classified into “dry” (0), “slight” (1–2), “moderate” (3–6), “severe” (8–9) and “very severe” (12). | They defined the MID using the ISI as the anchor as the difference in change in score between those subjects who had a one level improvement on ISI and those whose ISI category did not change from baseline (e.g. Severe to moderate, slight to dry, etc.). | Slight |
|  | Urogenital Distress Inventory (UDI) – Stress |  |  |  |  |  |
|  | UIQ |  |  |  |  |  |
|  | Urogenital Distress Inventory (UDI) | Voiding diary | ≥ 0.3 | They classified the answers from the bladder diary in “worse: ≥ 25% increase in number of incontinence episodies; “no change”: a change in any direction between 0 and 24%; and “better”: ≥ 25% decrease in the number of incontinence episodies | MID was defined according the difference in scores between those who were “better” and those who demonstrated “no change.” | Strong |
|  | Urogenital Distress Inventory (UDI) – Stress |  |  |  |  |  |
|  | UIQ |  |  |  |  |  |
| Yalcin et al.,(40) | Incontinence Quality of Life (I-QOL) – Within-treatment (mean, SD) – Total score | Patient Global Impression of Improvement questionnaire | Not reported | Seven possible answers were avaiable in the anchor: very much, much, and a little better, no change, and a little, much, and very much worse. | They compared participants that were “a little better” and women the reported “no change” in their conditions | Slight |
|  | Incontinence Quality of Life (I-QOL) – Within-treatment (mean, SD) – Avoidance and Limiting Behavior |  |  |  |  |  |
|  | Incontinence Quality of Life (I-QOL) – Within-treatment (mean, SD) – Psychosocial Impacts |  |  |  |  |  |
|  | Incontinence Quality of Life (I-QOL) – Within-treatment (mean, SD) – Social Embarassement |  |  |  |  |  |
| Chan et al.,(43) | UIQ subscale of PFIQ | Self-reported satisfaction with the treatment received (very satisfied, moderately satisfied, somewhat satisfied or dissatisfied) | Not reported | Participants should answer if they were very satisfied, moderately satisfied, somewhat satisfied or dissatisfied with the treatment | The difference in the mean change score for the women who were “somewhat satisfied” and the mean change score for the “dissatisfied” group were used to calculate the MID | Slight |
|  | UDI subscale of PFDI |  |  |  |  |  |
|  | UIQ subscale of PFIQ | 10-cm VAS score indicating the severity of symptoms | 0,56 | A 10-cm scale was used to analyzed the severity of the symptom | Authors considered the change in VAS score >0 for the “getting worse” group, a change of −2 to 0 for the “no change/slightly improved” group, a change of −4 to −2.1 for the “improved” group, and a change of <−4.1 for the “greatly improved” group. The MID was defined as the difference in mean change score for the women who were “improved” and the mean change score for the “no change/slightly improved” group | Slight |
|  | UDI subscale of PFDI |  |  |  |  |  |
| Dyer et al.,(58) | Urogenital Distress Inventory (UDI) | Voiding diary – 25% | ≥ 0.3 | The cut-off for characterizing the population was the decrease in (I) ≥25% and (II) ≥75% in the reduction in incontinence episodes on the 7-day voiding diary. | The calculation was based on: improved (≥25% decrease in incontinence episodies), no change (0 to 25%) and worse (≥25% increase in the incontinence episodies). The same calculation was performed considering the cut-off of 75% in the decrease of urinary episodies | Strong |
|  | UDI – Irritative Subscale |  |  |  |  |  |
|  | Overactive Bladder Questionnaire (OAB-q) – Symptom Severity |  |  |  |  |  |
|  | Urogenital Distress Inventory (UDI) | Global Perception of Improvement | ≥ 0.3 | The anchor should be filled according to the following options: much better, better, about the same, worse or much worse | They used the difference in mean questionnaire scores between patients reporting “better” and those reporting “about the same” during data analysis | Moderate |
|  | UDI – Irritative Subscale |  |  |  |  |  |
|  | Overactive Bladder Questionnaire (OAB-q) – Symptom Severity |  |  |  |  |  |
|  | Urogenital Distress Inventory (UDI) | Patient Satisfaction Questionnaire | ≥ 0.3 | Participants should complete the anchor according to the following options: completely satisfied, somewhat satisfied, or not at all satisfied | The difference in mean questionnaire scores between patients reporting “somewhat satisfied” and those reporting “not at all” was used during data analysis | Strong |
|  | UDI – Irritative Subscale |  |  |  |  |  |
|  | Overactive Bladder Questionnaire (OAB-q) – Symptom Severity |  |  |  |  |  |
| Lim et al.,(48) | International Consultation on Incontinence Questionnaire – Short Form (ICIQ-SF) | Patient Global Impression of Improvement questionnaire | ≥ 0.3 | Seven levels of answers were avaiable: very much better, much better, a little better, no change, a little worse, much worse, very much worse | The difference in the mean ICIQ-UI-SF scores between participants who reported “very much better” or “much better” vs all other responses was used to calculate MID | Strong |
|  | ICIQ-Lower Urinary Tract Symptoms Quality of Life (ICIQ-LUTSqol) |  |  |  |  |  |
|  | International Consultation on Incontinence Questionnaire – Short Form (ICIQ-SF) | Satisfaction with the treatment | ≥ 0.3 | To fill the anchor, the following answers were avaiable: completely satisfied, mostly satisfied, neutral, mostly dissatisfied, completely dissatisfied) | The difference in the mean ICIQ-UI SF scores between “positive” (completely satisfied or mostly satisfied) and “neutral or negative” (neutral or mostly dissatisfied or completely dissatisfied) response was used to calculate MID | Strong |
|  | ICIQ-Lower Urinary Tract Symptoms Quality of Life (ICIQ-LUTSqol) |  |  |  |  |  |
|  | International Consultation on Incontinence Questionnaire – Short Form (ICIQ-SF) | 1-h pad test | ≥ 0.3 | Authors used the cut-off point of 50% of reduction in the incontinence episodes | The difference in the mean ICIQ-UI-SF scores between patients with a ≥50% reduction in the objective measures (1-hour pad test or voiding diary) and those with less than 50% reduction (considered ad the group that presented no change or was worse) was used | Strong |
|  | ICIQ-Lower Urinary Tract Symptoms Quality of Life (ICIQ-LUTSqol) |  |  |  |  |  |
|  | International Consultation on Incontinence Questionnaire – Short Form (ICIQ-SF) | Voiding diary | ≥ 0.3 | Authors used the cut-off point of 50% of reduction in the incontinence episodes | The difference in the mean ICIQ-UI-SF scores between patients with a ≥50% reduction in the objective measures (1-hour pad test or voiding diary) and those with less than 50% reduction (considered ad the group that presented no change or was worse) was used | Strong |
|  | ICIQ-Lower Urinary Tract Symptoms Quality of Life (ICIQ-LUTSqol) |  |  |  |  |  |
| Nystrom et al.,(45) | International Consultation on Incontinence Questionnaire – Short Form (ICIQ-SF) | Patient Global Impression of Improvement questionnaire | Not reported | Participants should fill the anchor considering their status as : very much better, much better, a little better, no change, a little worse, much worse and very much worse.’ | They calculated the MID according to patients that were “a little better” and “no change” | Slight |
|  | ICIQ-Lower Urinary Tract Symptoms Quality of Life (ICIQ-LUTSqol) |  |  |  |  |  |
| Patrick et al.,(39) | Incontinence Quality of Life (I-QOL) | Patient Global Perception of Change | Not reported | The options to fill the anchor were: very much better, much better, a little better, the same, a little worse, worse, much worse | In methods they cited “Worse, the same, better”, but in the results section they said that those who reported a “little better” condition presented 2 point of improvement in I-QOL | Slight |
|  | Incontinence Quality of Life (I-QOL) | Pad Test | Not reported | Authors considered the following cut-off to calculate MID: worse: 5-25% increased in pad-test or incontinence epidoes according to the voiding diary: worse; same or no change: a change in any direction between 0 and 24%; better: 5 a 25% or greater decrease in pad-test number of incontinent episodes | It is not clear which group of comparison was used in the analysis | Strong |
|  | Incontinence Quality of Life (I-QOL) | Voiding diary | Not reported | Authors considered the following cut-off to calculate MID: worse: 5-25% increased in pad-test or incontinence epidoes according to the voiding diary: worse; same or no change: a change in any direction between 0 and 24%; better: 5 a 25% or greater decrease in pad-test number of incontinent episodes | It is not clear which group of comparison was used in the analysis | Strong |
| Sirls et al.,(46) | International Consultation on Incontinence Questionnaire - Short Form (ICIQ-SF) | Patient Global Impression of Improvement questionnaire | ≥ 0.3 | Options to fill the anchor were: very much better, much better, a little better, no change, a little worse, much worse, very much worse | Authors used the difference in mean ICIQ-UI SF scores between patients reporting “very much better” and all other responses | Strong |
|  |  | Satisfaction with the treatment | ≥ 0.3 | The options to fill the anchor were: completely satisfied, mostly satisfied, neutral, mostly dissatisfied, completely dissatisfied | They compared “somewhat satisfied (completely satisfied or mostly satisfied or neutral)” and those reporting “not at all satisfied (mostly dissatisfied or completely dissatisfied) | Strong |
|  |  | Voiding diary - 25% | ≥ 0.3 | Authors performed two calculations: first considering the cut-off point of 25%, and sequentially the cut-off of 75%. The following options were included: worse: >=25%/75% increase incontinence episodes, 0-25%/75%: no change, better: >=25%/75% decrease in incontinence episodes | The statistical analysis was performed between those who were “better” and those who demonstrated “no change” | Strong |
|  |  | Urogenital Distess Inventory (UDI) | ≥ 0.3 | Authors considered a ≥75% reduction in the urinary symptoms to allocated subjects in three different groups: improved (≥ 75% decrease), no change (0 to 75%) and worse: (≥ 75% increase) | They compared the difference in ICIQ-UI SF scores between patients with a ≥75% reduction versus no change | Strong |
|  |  | Incontinence Impact Questionnaire (IIQ) | ≥ 0.3 | Authors considered a ≥75% reduction in the urinary symptoms to allocated subjects in three different groups: improved (≥ 75% decrease), no change (0 to 75%) and worse: (≥ 75% increase) | They compared the difference in ICIQ-UI SF scores between patients with a ≥75% reduction versus no change | Strong |
| Baessler et al.,(49) | Australian Pelvic Floor Questionnaire - Bladder | Patient Global Impression of Improvement questionnaire | ≥ 0.3 | The PGI-I ranging from -3 to +3 was used during data collection. No further details were provided by the authors. | The MID was calculated as the difference in score changes between women with PGI-I= 0 and PGI-I=1 in the bladder domain for the SUT group | Slight |
|  | Australian Pelvic Floor Questionnaire – Global score |  |  |  |  |  |
| Nipa et al., (50) | ICIQ-FLUTS: International Consultation on Incontinence Questionnaire – Female Lower Urinary Tract Symptoms | Patient Global Impression of Improvement questionnaire | Not reported | MID was obtained by comparing the group of participants responding ‘improved’, classed as success, to the PGI-I to the group of participants responding ‘same’, classed as failure. Participants responding ‘much improved’, ‘very much improved’, ‘worse’, ‘much worse’ and ‘very much worse’ were not included in this analysis | | Slight |
|  | International Consultation on Incontinence Questionnaire - Short Form (ICIQ-SF) |  | Not reported |  |  |  |
