# Supplementary material for: The minimal important difference of patient-reported outcome measures related to female urinary incontinence: a systematic review
Source: BMC Med Res Methodol. 2024 Mar 8;24:60. doi: 10.1186/s12874-024-02188-4 (PMC10921720; doi:10.1186/s12874-024-02188-4)
Supplement: Supplementary file 8 — Supplementary Material 8. [file 12874_2024_2188_MOESM8_ESM.docx]

**Appendix 8**. Matrix regarding the data extracted from the studies included in the present systematic review, considering distribution- and anchor based-methods.

| Distribution based-methods | | | | | | | | | | | | | | | | | | | | | | | |  | |
| --- | --- | --- | --- | --- | --- | --- | --- | --- | --- | --- | --- | --- | --- | --- | --- | --- | --- | --- | --- | --- | --- | --- | --- | --- | --- |
| PROM | **Analysis** | Barber et al., 2009(41) | | Yalcin et al., 2005(40) | | Chan et al., 2013(43) | | Dyer et al., 2011(42) | | Lim et al., 2019(48) | | Luz et al, 2017(47) | | Nystrom et al., 2015(45) | | Patrick et al., 1999(39) | | Sirls et al., 2015(46) | | Suskind et al., 2014(44) | | Baessler et al., 2019(49) | |  | |
| Australian Pelvic Floor Questionnaire | Effect size | - | | - | | - | | - | | - | | - | | - | | - | | - | | - | | X | |  | |
|  | Standardized response mean | - | | - | | - | | - | | - | | - | | - | | - | | - | | - | | X | |  | |
| Incontinence Impact Questionnaire (IIQ) | Effect size | - | | - | | - | | X | | - | | - | | - | | - | | - | | - | | - | |  | |
|  | Standard error of measurement | - | | - | | - | | X | | - | | - | | - | | - | | - | | - | | - | |  | |
| International Consultation on Incontinence Questionnaire - Short Form (ICIQ-SF) | Effect size | - | | - | | - | | - | | X | | - | | - | | - | | X | | - | | - | |  | |
| International Consultation on Incontinence Questionnaire -Lower Urinary Tract Symptoms Quality of Life (ICIQ-LUTSqol) | Effect size | - | | - | | - | | - | | X | | - | | - | | - | |  | | - | | - | |  | |
| King’s Health Questionnaire (KHQ) | Effect size | - | | - | | - | | - | | - | | X | | - | | - | | - | | - | | - | |  | |
| Michigan Incontinence Symptom Index (M-ISI) | Effect size | - | | - | | - | | - | | - | | - | | - | | - | | - | | X | | - | |  | |
| Urogenital Distress Inventory (UDI) | Effect size | X | | - | | X | | X | | - | | - | | - | | - | | - | | - | | - | |  | |
|  | Standard error of measurement | X | | - | | X | | X | | - | | - | | - | | - | | - | | - | | - | |  | |
| Urogenital Distress Inventory (UDI-Irritative Subscale) | Effect size | - | | - | | - | | - | | X | | - | | - | | - | | - | | - | | - | |  | |
|  | Standard error of measurement | X | | - | | - | | X | | - | | - | | - | | - | | - | | - | | - | |  | |
| Urinary Distress Inventory (UDI-stress) | Effect size | X | | - | | - | | - | | - | | - | | - | | - | | - | | - | | - | |  | |
|  | Standard error of measurement | X | | - | | - | | - | | - | | - | | - | | - | | - | | - | | - | |  | |
| Urinary Impact Questionnaire (UIQ) | Effect size | X | | - | | X | | - | | - | | - | | - | | - | | - | | - | | - | |  | |
|  | Standard error of measurement | X | | - | | X | | - | | - | | - | | - | | - | | - | | - | | - | |  | |
| Overactive Bladder Questionnaire (OAB-q) | Effect size | - | | - | | - | | X | | - | | - | | - | | - | | - | | - | | - | |  | |
|  | Standard error of measurement | - | | - | | - | | X | | - | | - | | - | | - | | - | | - | | - | |  | |
| Overactive Bladder Questionnaire (OAB-q- Symptom severity) | Standard error of measurement | - | | - | | - | | X | | - | | - | | - | | - | | - | | - | | - | |  | |
|  | Effect size | - | | - | | - | | X | | - | | - | | - | | - | | - | | - | | - | |  | |
| Anchor based-methods | | | | | | | | | | | | | | | | | | | | | | | |  | |
| PROM | **Anchor** | Barber et al., 2009(41) | Yalcin et al, 2005(40) | | Chan et al., 2013(43) | | Dyer et al., 2011(42) | | Lim et al., 2019(48) | | Luz et al., 2017(47) | | Nystrom et al., 2015(45) | | Patrick et al., 1999(39) | | Sirls et al., 2015(46) | | Baessler et al., 2019(49) | | Suskind et al., 2014(44) | | Nipa et al., 2023(50) | |  |
| Australian Pelvic Floor Questionnaire | Patient Global Impression of Improvement questionnaire | - | | - | | - | | - | | - | | - | | - | | - | | - | | X¶ | | - | |  | |
| International Consultation on Incontinence Questionnaire - Short Form (ICIQ-SF) | Patient Global Impression of Improvement questionnaire | - | | - | | - | | - | | X▲ | | - | | X¶ | | - | | X▲ | | - | | X¶ | |  | |
|  | Satisfaction with the treatment | - | | - | | - | | - | | X▲ | | - | | - | | - | | X▲ | | - | | - | |  | |
|  | Voiding diary | - | | - | | - | | - | | X▲ | | - | | - | | - | | X§▲ | | - | | - | |  | |
|  | Pad test | - | | - | | - | | - | | X▲ | | - | | - | | - | |  | | - | | - | |  | |
|  | Urogenital Distress Inventory (UDI) | - | | - | | - | | - | | - | | - | | - | | - | | X▲ | | - | | - | |  | |
|  | Incontinence Impact Questionnaire (IIQ) | - | | - | | - | | - | | - | | - | | - | | - | | X▲ | | - | | - | |  | |
| ICIQ-Lower Urinary Tract Symptoms Quality of Life (ICIQ-LUTSqol) | Patient Global Impression of Improvement questionnaire | - | | - | | - | | - | | X▲ | | - | | X¶ | | - | | - | | - | | - | |  | |
|  | Satisfaction with the treatment | - | | - | | - | | - | | X▲ | | - | | - | | - | | - | | - | | - | |  | |
|  | Voiding diary | - | | - | | - | | - | | X▲ | | - | | - | | - | | - | | - | | - | |  | |
|  | Pad test | - | | - | | - | | - | | X▲ | | - | | - | | - | | - | | - | | - | |  | |
| Incontinence Quality of Life (I-QOL) | Patient Global Impression of Improvement questionnaire | - | | X¶ | | - | | - | | - | | - | | - | | X¶▲ | | - | | - | | - | |  | |
|  | Voiding diary | - | | - | | - | | - | | - | | - | | - | | X▲ | | - | | - | | - | |  | |
|  | Pad test | - | | - | | - | | - | | - | | - | | - | | X▲ | | - | | - | | - | |  | |
| Urogenital Distress Inventory (UDI) | Patient Global Impression of Improvement questionnaire | X¶ | | - | | - | | - | | - | | - | | - | | - | | - | | - | | - | |  | |
|  | Global Perception of Improvement | - | | - | | - | | X§ | | - | | - | | - | | - | | - | | - | | - | |  | |
|  | Patient Satisfaction Questionnaire | - | | - | | - | | X§ | | - | | - | | - | | - | | - | | - | | - | |  | |
|  | Incontinence Severity Index | X€ | | - | | - | |  | | - | | - | | - | | - | | - | | - | | - | |  | |
|  | Voiding diary | X¶ | | - | | - | | X¶▲ | | - | | - | | - | | - | | - | | - | | - | |  | |
|  | Satisfaction with the treatment | - | | - | | X¶ | | - | | - | | - | | - | | - | | - | | - | | - | |  | |
|  | 10-cm VAS score indicating the severity of symptoms | - | | - | | X¶ | | - | | - | | - | | - | | - | | - | | - | | - | |  | |
| Urogenital Distress Inventory (UDI-Irritative Subscale) | Global Perception of Improvement | - | | - | | - | | X§ | | - | | - | | - | | - | | - | | - | | - | |  | |
|  | Patient Satisfaction Questionnaire | - | | - | | - | | X§ | | - | | - | | - | | - | | - | | - | | - | |  | |
|  | Voiding diary | - | | - | | - | | X¶▲ | | - | | - | | - | | - | | - | | - | | - | |  | |
| Urogenital Distress Inventory (UDI-stress) | Patient Global Impression of Improvement questionnaire | X¶ | | - | | - | | - | | - | | - | | - | | - | | - | | - | | - | |  | |
|  | Voiding diary | X¶ | | - | | - | | - | | - | | - | | - | | - | | - | | - | | - | |  | |
|  | Incontinence Severity Index | X€ | | - | | - | | - | | - | | - | | - | | - | | - | | - | | - | |  | |
| Urinary Impact Questionnaire (UIQ) | Patient Global Impression of Improvement questionnaire | X¶ | | - | | - | | - | | - | | - | | - | | - | | - | | - | | - | |  | |
|  | Voiding diary | X¶ | | - | | - | | - | | - | | - | | - | | - | | - | | - | | - | |  | |
|  | Incontinence Severity Index | X€ | | - | | - | | - | | - | | - | | - | | - | | - | | - | | - | |  | |
|  | Satisfaction with the treatment | - | | - | | X¶ | | - | | - | | - | | - | | - | | - | | - | | - | |  | |
|  | 10-cm VAS score indicating the severity of symptoms | - | | - | | X¶ | | - | | - | | - | | - | | - | | - | | - | | - | |  | |
| Overactive Bladder Questionnaire ((58)) | Global Perception of Improvement | - | | - | | - | | X§ | | - | | - | | - | | - | | - | | - | | - | |  | |
|  | Patient Satisfaction Questionnaire | - | | - | | - | | X§ | | - | | - | | - | | - | | - | | - | | - | |  | |
|  | Voiding diary | - | | - | | - | | X¶▲ | | - | | - | | - | | - | | - | | - | | - | |  | |
| International Consultation on  Incontinence Questionnaire – Female Lower Urinary Tract  Symptoms (ICIQ-FLUTS) | Patient Global Impression of Improvement questionnaire | - | | - | | - | | - | | - | | - | | - | | - | | - | | - | | X¶ | |  | |

PROM: patient reported outcome; VAS: visual analogue scale

¶: MID calculated according to a slight improvement from the anchor; §: MID calculated according to a moderate improvement from the anchor; ▲: MID calculated according to a strong improvement from the anchor; €: the calculations were based on 1 point of difference for each level of severity that had changed (very severe to severe, severe to moderate, moderate to slight)
