# Supplementary material for: The minimal important difference of patient-reported outcome measures related to female urinary incontinence: a systematic review
Source: BMC Med Res Methodol. 2024 Mar 8;24:60. doi: 10.1186/s12874-024-02188-4 (PMC10921720; doi:10.1186/s12874-024-02188-4)
Supplement: Supplementary file 9 — Supplementary Material 9. [file 12874_2024_2188_MOESM9_ESM.docx]

**Appendix 6**. GRADE assessment.

| Nº | PROM | Nº of studies | Nº of individuals | Study design | Risk of bias | Inconsistency | Indirectness | Imprecision* | Other considerations | Overall quality |
| --- | --- | --- | --- | --- | --- | --- | --- | --- | --- | --- |
| 1.1  1.2 | Australian Pelvic Floor Questionnaire | 1(1) | 80 | cross-sectional | very serious^a^ | serious^b^ | not serious | very serious^c^ | none | ⨁◯◯◯ Very low |
| 1.3  1.4  1.8  1.9 | IQOL – Total score | 2(2, 3) | 1421 | randomised trials | very serious^a^ | very serious^d,e^ | very serious^f^ | not serious | none | ⨁◯◯◯ Very low |
| 1.5  1.6  1.7 | IQOL - Subscales | 1(3) | 1133 | randomised trials | very serious^a^ | serious^b^ | not serious | not serious | none | ⨁◯◯◯ Very low |
| 1.10-1.20 | ICIQ-SF | 4(4, 5, 6, 7) | 821 | randomised trials | very serious^a^ | very serious^d,f,g^ | very serious^f^ | not serious | none | ⨁◯◯◯ Very low |
| 1.25-1.29 | ICIQ-LUTSqol | 2(4, 5) | 320 | randomised trials | very serious^a^ | serious^f,g^ | very serious^f^ | not serious | none | ⨁◯◯◯ Very low |
| 1.30-1.34 | UIQ | 2(8, 9, 10) | 400 | randomised trials, clinical trial | very serious^a^ | serious^d^ | not serious | not serious | none | ⨁◯◯◯ Very low |
| 1.35-1.46 | UDI | 3(10, 11) | 672 | randomised trials | very serious^a^ | very serious^d,f,g^ | serious^f^ | not serious | none | ⨁◯◯◯ Very low |
| 1.47-1.54 | UDI – Irritative subscale | 1(11) | 272 | randomised trials | very serious^a^ | serious^b^ | serious^f^ | serious^c^ | none | ⨁◯◯◯ Very low |
| 1.55-1.57 | UDI – Stress subscale | 1(10) | 333 | randomised trials | very serious^a^ | serious^b^ | serious^f^ | not serious | none | ⨁◯◯◯ Very low |
| 1.58-1.65 | OAB-q | 1(11) | 272 | randomised trials | very serious^a^ | serious^b^ | serious^f^ | serious^c^ | none | ⨁◯◯◯ Very low |
| 1.73-1.78 | ICIQ‑FLUTS | 1(7) | 912 | randomised trials | very serious^a^ | serious^b,g^ | not serious | not serious | none | ⨁◯◯◯ Very low |

#### ICIQ-SF: International Consultation on Incontinence Questionnaire - Short Form; I-QOL: Incontinence Quality of Life; OAB-q: Overactive bladder questionnaire; PROM: patient-reported outcome measure; UDI: Urogenital Distress Inventory; UIQ: Urinary Impact Questionnaire

#### *the cut-off to determine imprecision was set in 300 individuals

#### Explanations

a. PROM was classified with low credibility by the MID credibility tool

b. There was only one study measuring this PROM, the effects were not estimated but the evidence was downgraded since there is a lack of studies measuring this outcome, which could be an indicator that the literature is not well established in the area

c. Sample size is considered small

d. Different anchors were applied in order to calculate MID

e. There are differences related to the population diagnosis

f. MIDs were provided according to different criteria (minimal, moderate or strong level of improvement)

g. Time-points differ across studies

**References**

1. Baessler K, Mowat A, Maher CF. The minimal important difference of the Australian Pelvic Floor Questionnaire. International Urogynecology Journal. 2019;30(1):115-22.

2. Patrick DL, Martin ML, Bushnell DM, Yalcin I, Wagner TH, Buesching DP. Quality of life of women with urinary incontinence: further development of the incontinence quality of life instrument (I-QOL). Urology. 1999;53(1):71-6.

3. Yalcin I, Patrick DL, Summers K, Kinchen K, Bump RC. Minimal clinically important differences in Incontinence Quality-of-Life scores in stress urinary incontinence. Urology. 2006;67(6):1304-8.

4. Lim R, Liong ML, Lim KK, Leong WS, Yuen KH. The Minimum Clinically Important Difference of the International Consultation on Incontinence Questionnaires (ICIQ-UI SF and ICIQ-LUTSqol). Urology. 2019;133:91-5.

5. Nystrom E, Sjostrom M, Stenlund H, Samuelsson E. ICIQ symptom and quality of life instruments measure clinically relevant improvements in women with stress urinary incontinence. Neurourology and urodynamics. 2015;34(8):747-51.

6. Sirls LT, Tennstedt S, Brubaker L, Kim HY, Nygaard I, Rahn DD, et al. The minimum important difference for the International consultation on incontinence questionnaire - Urinary incontinence short form in women with stress urinary incontinence. Neurourology and Urodynamics. 2015;34(2):183-7.

7. Nipa SO, Cooper D, Mostada A, Hagen S, Abdel-Fattah M. Novel clinically meaningful scores for the ICIQ‑UI‑SF and ICIQ‑FLUTS questionnaires in women with stress incontinence. International urogynecology journal. 2023;34:3033-40.

8. Barber MD, Spino C, Janz NK, Brubaker L, Nygaard I, Nager CW, et al. The minimum important differences for the urinary scales of the Pelvic Floor Distress Inventory and Pelvic Floor Impact Questionnaire. American Journal of Obstetrics and Gynecology. 2009;200(5):580.e1-.e7.

9. Chan SSC, Cheung RYK, Lai BPY, Lee LL, Choy KW, Chung TKH. Responsiveness of the Pelvic Floor Distress Inventory and Pelvic Floor Impact Questionnaire in women undergoing treatment for pelvic floor disorders. International Urogynecology Journal and Pelvic Floor Dysfunction. 2013;24(2):213-21.

10. Barber MD, Spino C, Janz NK, Brubaker L, Nygaard I, Nager CW, et al. The minimum important differences for the urinary scales of the Pelvic Floor Distress Inventory and Pelvic Floor Impact Questionnaire. American Journal of Obstetrics and Gynecology. 2009;200(5).

11. Dyer KY, Xu Y, Brubaker L, Nygaard I, Markland A, Rahn D, et al. Minimum important difference for validated instruments in women with urge incontinence. Neurourology and Urodynamics. 2011;30(7):1319-24.
